# New Heterocyclic Compounds from Oxazol-5(4*H*)-one and 1,2,4-Triazin-6(5*H*)-one Classes: Synthesis, Characterization and Toxicity Evaluation

**DOI:** 10.3390/molecules28124834

**Published:** 2023-06-17

**Authors:** Stefania-Felicia Barbuceanu, Elena-Valentina Rosca, Theodora-Venera Apostol, Laura-Ileana Socea, Constantin Draghici, Ileana Cornelia Farcasanu, Lavinia Liliana Ruta, George Mihai Nitulescu, Lucian Iscrulescu, Elena-Mihaela Pahontu, Rica Boscencu, Gabriel Saramet, Octavian Tudorel Olaru

**Affiliations:** 1 Department of Organic Chemistry, Faculty of Pharmacy, “Carol Davila” University of Medicine and Pharmacy, 6 Traian Vuia Street, 020956 Bucharest, Romania; elenavalentinarosca90@gmail.com (E.-V.R.); laura.socea@umfcd.ro (L.-I.S.); lucian.iscrulescu@umfcd.ro (L.I.); 2 “C. D. Nenitescu” Institute of Organic and Supramolecular Chemistry Romanian Academy, 202B Splaiul Independenței, 060023 Bucharest, Romania; cst.drag@yahoo.com; 3 Department of Organic Chemistry, Biochemistry and Catalysis, Faculty of Chemistry, University of Bucharest, 90–92 Panduri Str., 050663 Bucharest, Romania; ileana.farcasanu@chimie.unibuc.ro (I.C.F.); lavinia.ruta@chimie.unibuc.ro (L.L.R.); 4 Department of Pharmaceutical Chemistry, Faculty of Pharmacy, “Carol Davila” University of Medicine and Pharmacy, 6 Traian Vuia Street, 020956 Bucharest, Romania; george.nitulescu@umfcd.ro; 5 Department of General and Inorganic Chemistry, Faculty of Pharmacy, “Carol Davila” University of Medicine and Pharmacy, 6 Traian Vuia Street, 020956 Bucharest, Romania; elena.pahontu@umfcd.ro (E.-M.P.); rica.boscencu@umfcd.ro (R.B.); 6 Department of Pharmaceutical Technology and Biopharmacy, Faculty of Pharmacy, “Carol Davila” University of Medicine and Pharmacy, 6 Traian Vuia Street, 020956 Bucharest, Romania; gabriel.saramet@umfcd.ro; 7 Department of Pharmaceutical Botany and Cell Biology, Faculty of Pharmacy, “Carol Davila” University of Medicine and Pharmacy, 6 Traian Vuia Street, 020956 Bucharest, Romania; octavian.olaru@umfcd.ro

**Keywords:** oxazol-5(4*H*)-one, 1,2,4-triazin-6(5*H*)-one, condensation, toxicity, *Daphnia magna*, *Saccharomyces cerevisiae*

## Abstract

This paper describes the synthesis of new heterocycles from oxazol-5(4*H*)-one and 1,2,4-triazin-6(5*H*)-one classes containing a phenyl-/4-bromophenylsulfonylphenyl moiety. The oxazol-5(4*H*)-ones were obtained via condensation of 2-(4-(4-X-phenylsulfonyl)benzamido)acetic acids with benzaldehyde/4-fluorobenzaldehyde in acetic anhydride and in the presence of sodium acetate. The reaction of oxazolones with phenylhydrazine, in acetic acid and sodium acetate, yielded the corresponding 1,2,4-triazin-6(5*H*)-ones. The structures of the compounds were confirmed using spectral (FT-IR, ^1^H-NMR, ^13^C-NMR, MS) and elemental analysis. The toxicity of the compounds was evaluated on *Daphnia magna* Straus crustaceans and on the budding yeast *Saccharomyces cerevisiae*. The results indicate that both the heterocyclic nucleus and halogen atoms significantly influenced the toxicity against *D. magna*, with the oxazolones being less toxic than triazinones. The halogen-free oxazolone had the lowest toxicity, and the fluorine-containing triazinone exhibited the highest toxicity. The compounds showed low toxicity against yeast cells, apparently due to the activity of plasma membrane multidrug transporters Pdr5 and Snq2. The predictive analyses indicated an antiproliferative effect as the most probable biological action. The PASS prediction and CHEMBL similarity studies show evidence that the compounds could inhibit certain relevant oncological protein kinases. These results correlated with toxicity assays suggest that halogen-free oxazolone could be a good candidate for future anticancer investigations.

## 1. Introduction

**Figure 1:**
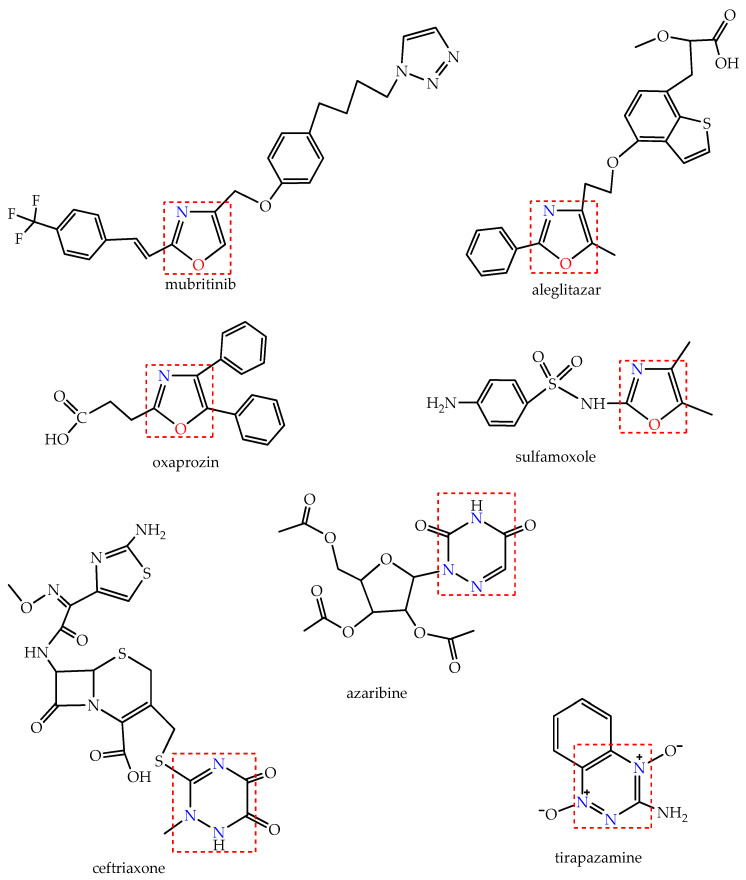
Structures of some drugs with an oxazole or 1,2,4-triazine nucleus.

The necessity of discovering new drugs that are more active and less toxic than those in use has boosted the synthesis of an increasing number of heterocyclic compounds. More than 85% of biologically active compounds possess a heterocyclic structure, and the vast majority of these contain nitrogen as a heteroatom [1,2]. The chemistry of heterocycles represents the most complex branch of organic and medicinal chemistry. Many aspects related to the structure, reactivity, synthesis and physico-chemical properties of heterocycles have captivated researchers, with an ultimate focus on revealing their biological activities and applications in various fields, including medicine, agriculture and industry [3]. Most of the known heterocycles are of natural origin, serving as a backbone in biologically active natural products used as traditional medication, or are synthetic compounds, many of them being subunits of active substances in the composition of various market drugs [2]. Among them, five-membered heterocyclic compounds from the 1,3-oxazole class have earned a distinct place in medicinal and pharmaceutical chemistry as a promising class in drug design. Oxazole is recognized as an efficient pharmacophore, being present in the structure of the active substances of some drugs, such as mubritinib (tyrosine kinase inhibitor) with antitumoral activity, oxaprozine (COX-2 inhibitor) or ditazole (platelet aggregation inhibitor) with anti-inflammatory activity, sulfamoxole (antibacterial) and aleglitazar (antidiabetic) [4,5,6] (Figure 1). Oxazolones in particular, depending on the position of the carbonyl group and the double bond, occur in five isomeric forms, the most important and best-studied being oxazol-5(4*H*)-ones. These compounds, also known as azlactones, are classified as saturated or unsaturated, according to the type of exocyclic bond linked to the carbon from four position of this core [7]. Unsaturated oxazolones are important synthons for the synthesis of different compounds with vast biological potential, including amino acids, peptides, amides [8,9,10,11,12] and five- or six-membered heterocycles (imidazolones, tiazolones, triazinones, etc.) [7,8,9,12]. A great number of 4-aryliden-oxazol-5(4*H*)-ones have a broad spectrum of biological activities that include antitumor [13,14], antibacterial [13,15,16,17], antifungal [13,15,16,17], anti-inflammatory [18,19], antioxidant [10,16,20] and antidiabetic [21] properties. 

On the other hand, triazines are important members of the class of six-membered heterocyclic compounds, because this versatile scaffold is found in many biologically active molecules with widespread applications. They can be used, for instance, as drugs for treatment of various diseases [22,23]. Out of the three isomeric forms of triazine, 1,2,4-triazines generate the most interest for medicinal chemistry research due to the diversity of their chemistry and biological potential [23,24,25]. Drugs containing a 1,2,4-triazine moiety with different pharmacological properties are currently used in clinics and clinical trials, e.g., ceftriaxone (antibiotic), lamotrigine (anticonvulsant), azaribine (antiviral, antifungal), tirapazamine (anticancer agent) and vardenafil (for erectile dysfunction) [22,23,24,25,26] (Figure 1). In particular, 1,2,4-triazin-6(5*H*)-ones have been reported for their biological properties, including antitumoral [9,27,28], antibacterial [29], antifungal [29], anti-inflammatory [18] and anticonvulsant activity [30]. 

Diaryl sulfone is another potent pharmacofore present in the structure of various bioactive compounds with antitumoral [31], antibacterial [32,33], antifungal [33], antioxidant [33] and antiviral [34] activity. Moreover, there are drugs with a sulfonyl group used in therapy; for example, dapsone is a commercially available antibacterial drug used to treat leprosy and various other infectious and chronic inflammatory diseases, also having antitumoral activity [31,35].

Based on these literature data, and in continuation of our drug discovery research program [36,37,38,39], we synthesized and characterized a series of new heterocyclic compounds from oxazol-5(4*H*)-one and 1,2,4-triazin-6(5*H*)-one classes with a diaryl sulfone moiety. The compounds were screened for their toxicity against *Daphnia magna* Straus and *Saccharomyces cerevisiae* cells. The *D. magna* bioassay is a commonly used method for assessing the toxicity of natural and synthetic compounds, serving as a preliminary screening tool for estimating the biological activity [40]. Also, the *S. cerevisiae* bioassay is a versatile method for evaluating the toxic effects and multidrug resistance (MDR) of chemical compounds [41,42].

## 2. Results and Discussion

### 2.1. Chemistry

Unsaturated azlactones can be obtain using synthetic procedures such as Erlenmeyer reaction, Bergmann synthesis, the reaction of ketoacids with primary amides or other catalytic methods under different conditions, the most facile, convenient and efficient being the first indicated method [43].

**Scheme 1:**
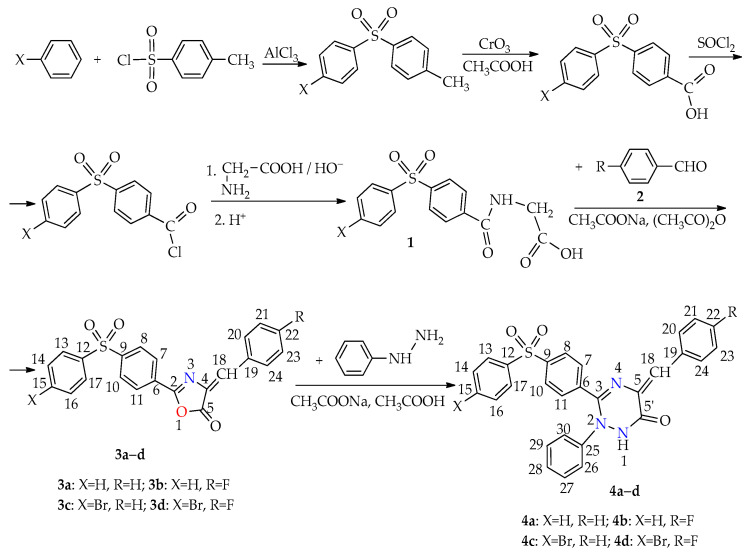
Synthesis of oxazol-5(4*H*)-ones **3a**–**d** and 1,2,4-triazin-6(5*H*)-ones **4a**–**d**.

The new oxazol-5(4*H*)-ones **3a**,**b**,**d** were synthesized according to the Erlenmeyer method [43], via the cyclocondensation of the 2-(4-(4-X-phenylsulfonyl)benzamido)acetic acids **1** [44,45] with benzaldehyde or 4-fluorobenzaldehyde **2**, under reflux, using acetic anhydride as a dehydrating agent and in the presence of sodium acetate, with 38–80% yields. The derivative **3c** has already been reported [38]. The new 1,2,4-triazin-6(5*H*)-ones **4a**–**d** were obtained via the condensation of the oxazolones **3a**–**d** with phenylhydrazine in acetic acid and in the presence of sodium acetate, under reflux, with 42–84% yields. The synthesis of key intermediates acids **1** was accomplished as previously described [44,45] via the N-acylation of glycine with 4-(4-X-phenylsulfonyl)benzoyl chlorides [46] that were prepared beginning with the alkylation of benzene or bromobenzene with *p*-toluenesulfonyl chloride, followed by oxidation of corresponding diaryl sulfones, and finally, the reaction of 4-(4-X-phenylsulfonyl)benzoic acids with thionyl chloride (Scheme 1). The structures of the newly synthesized compounds were established based on the recorded spectral data (IR, ^1^H-NMR, ^13^C-NMR and MS; see the Supplementary Materials).

The IR spectra of new oxazolones **3** confirmed the cyclocondensation of N-acylated glycine derivatives **1** with aldehydes through the disappearance of the NH absorption band from 3416 cm^−1^ (X = H) and 3341 cm^−1^ (X = Br) of these precursors [44,45]. The stretching vibration of the C=O lactone group from the oxazolones **3** is highlighted by a characteristic double absorption due to Fermi resonance, between 1771 and 1798 cm^−1^ [10,17,47]. In the IR spectra of new compounds **4a**–**d**, the appearance of a new absorption band in the range 3231–3331 cm^−1^, characteristic of stretching vibration of the NH group from the triazinone ring, confirms that the reaction of oxazolones **3a**–**d** with phenylhydrazine took place. Also, the absorption band from 1709 to 1720 cm^−1^ is due to stretching vibration of the C=O group from the triazinone ring. In the ^1^H-NMR spectra of the new compounds **3**, the most important proof that the condensation reaction occurred was the disappearance of the triplet signal due to the NH proton from precursors **1** (9.09 ppm when X = H [45] and 8.20 ppm when X = Br [44]) and the presence of a new singlet characteristic of the proton from the =CH group, with a chemical shift δ in the range of 7.43–7.46 ppm. In the case of compounds **4**, the new singlet signal characteristic of the NH proton from the triazinone ring appeared at δ = 8.98–9.00 ppm, and the =CH proton from the phenylhydrazine moiety resonated between 7.33 and 7.36 ppm as a singlet signal. The oxazolone ring is confirmed in the ^13^C-NMR spectra by the signals of the C-2 (161.5–161.7 ppm), C-4 (132.3–135.0 ppm) and C-5 (166.3–166.4 ppm) atoms. The signals of the C-3 and C-5 atoms from triazinone core are highlighted at δ values between 159.5 and 159.7 ppm and 133.7 and 135.7 ppm, respectively. The =CH carbon signal from oxazolones **3a**–**d** appeared at δ = 131.2–132.6 ppm and in triazinones **4a**–**d** in region 128.47–129.85 ppm. Also, the C=O carbon from triazinones resonated in the range 168.7–168.8 ppm. 

### 2.2. Toxicity Assays

#### 2.2.1. *Daphnia magna* Bioassay

**Figure 2:**
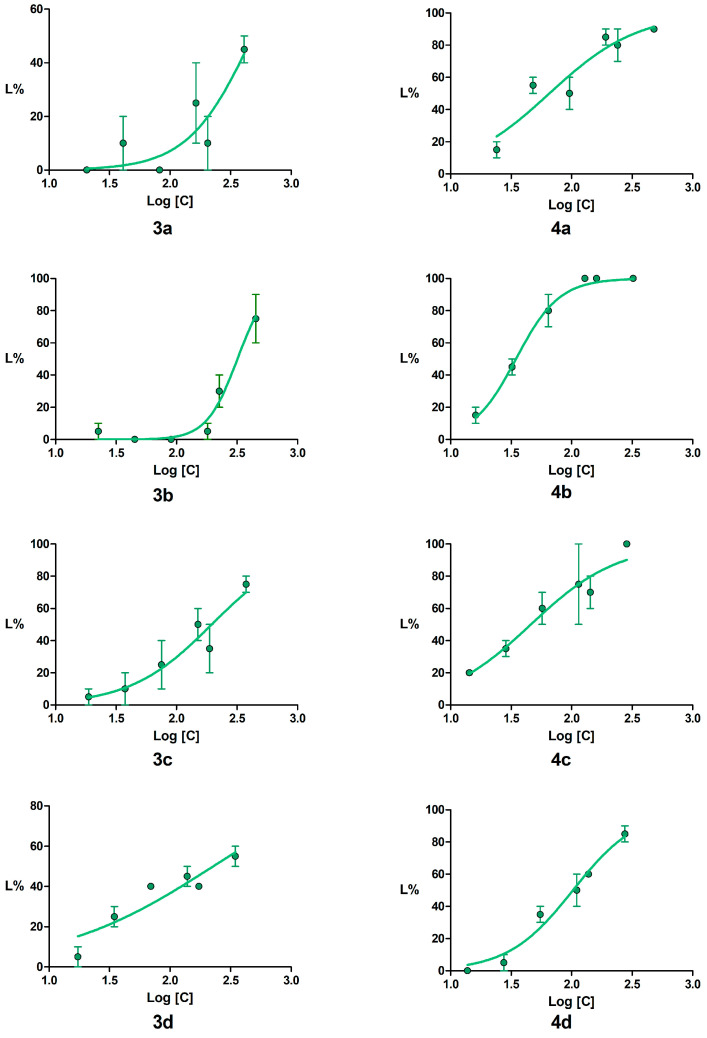
*D. magna* lethality curves after 48 h of exposure on compounds **3a**–**d** and **4a**–**d**. Values are mean ± SEM of duplicate determinations.

The results of the toxicity evaluation of oxazolones **3a**–**d** and triazinones **4a**–**d** on *Daphnia magna* are presented in Table 1 and Figure 2. After 24 h of exposure, the tested compounds induced a lethality of less than 30%, and therefore the LC_50_ could not be calculated. After 48 h of exposure, triazinones **4a**–**d** induced a higher toxicity compared to oxazolones **3a**–**d**. Of the oxazolone derivatives, compound **3a** induced the lowest toxicity. In the case of the other oxazolones, due to having a fluorine atom on the benzylidene fragment or a bromine atom on the phenylsulfonylphenyl moiety, or both halogens, the toxicity was increased. Among all tested compounds, triazinone **4b**, containing a fluorine atom on the arylidene fragment grafted to the 5 position of the heterocyclic core, was the most toxic. Triazinone derivatives **4c** and **4a** also induced a high toxicity, followed by **4d**, which showed a moderate to high action, suggesting a major effect of the triazinone nucleus, which was probably influenced by the halogen atom.

#### 2.2.2. *Saccharomyces cerevisiae* Toxicity Assay

**Figure 3:**
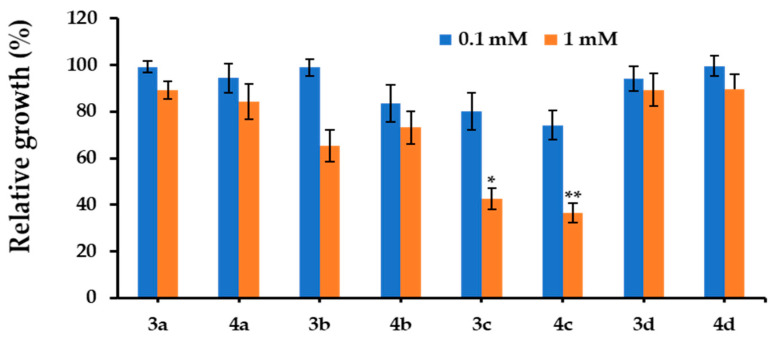
Effect of compounds **3a**–**d** and **4a**–**d** on the growth of *S. cerevisiae*. Early log phase BY4741 cells in YPD were treated with compounds **3a**–**d** and **4a**–**d** (0.1 mM or 1 mM final concentrations) and incubated at 28 °C with shaking (200 rpm) for 24 h. Cell growth was assessed spectrophotometrically (OD_600_) relative to cells grown in the absence of any supplemental compound. Values are mean ± SEM of triplicate determinations carried out on three biological repeats. * *p* < 0.05; ** *p* < 0.01.

The toxicity against the *S. cerevisiae* cells was evaluated by monitoring the cell’s proliferation when exposed to compounds **3a**–**d** and **4a**–**d**. The yeast cells were incubated for 24 h in rich YPD medium supplemented or 0.1 mM and 1 mM solutions of compounds **3a**–**d** or **4a**–**d**. Cell growth in the presence of each compound was calculated relative to the cell growth in the absence of any compound, but with an equivalent DMSO concentration. These two concentrations were set as the limits of the concentration range, since it was noted that compounds **3a**–**d** and **4a**–**d** were practically non-toxic at concentrations of less than 0.1 mM, while concentrations higher than 1 mM were cumbersome, causing precipitation in the incubation medium. The effect of compounds **3a**–**d** and **4a**–**d** (at 0.1 mM and 1 mM) on the growth of *S. cerevisiae* is presented in Figure 3.

As seen in Figure 3, the compounds exhibited low toxicity at a 0.1 mM concentration, and the growth of the yeast cells exposed to 0.1 mM **3a**–**d** or **4a**–**d** was not significantly different compared to the control. On the other hand, at 1 mM, the highest toxicity was noted for triazinone **4c**, followed by its corresponding precursor, oxazolone **3c**. At 1 mM, oxazolone **3c** reduced the growth of the yeast by 57.4% and triazinone **4c** reduced the growth by 63.5%, suggesting that introducing a bromine atom in the structure of these compounds but also the presence of this six-membered core (**4c**) might increase the toxicity.

**Figure 4:**
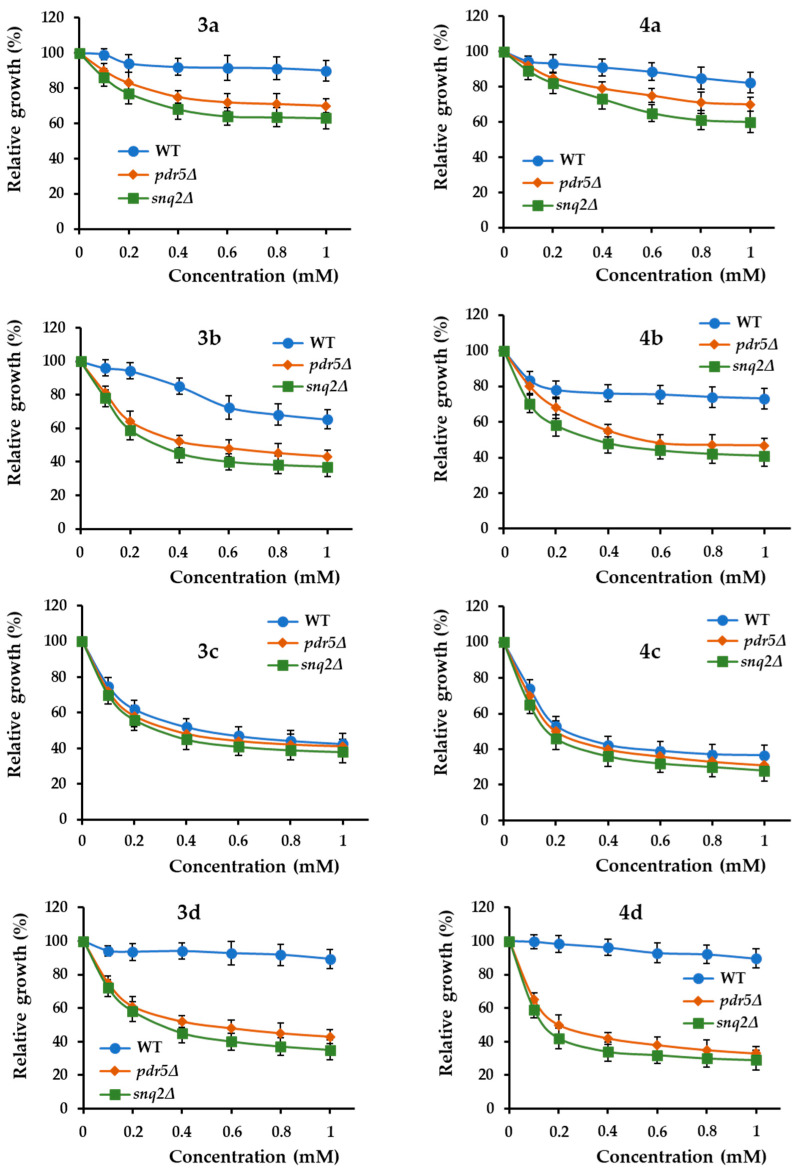
Effect of compounds **3a**–**d**/**4a**–**d** on the growth of *S. cerevisiae* strains defective in multidrug transporters Pdr5 or Snq2. WT—*S. cerevisiae* strains considered wild type. Early log phase BY4741 cells in YPD were treated with compounds **3a**–**d** and **4a**–**d** (0–1 mM final concentrations) and incubated at 28 °C with shaking (200 rpm) for 24 h. Cell growth was assessed spectrophotometrically (OD_600_) relative to cells grown in the absence of any supplemental compound. Values are mean ± SEM of triplicate determinations carried out on three biological repeats.

The least toxic compounds at either 0.1 mM (**3d**—94.2%, **4d**—99.6%) or 1 mM (**3d**—89.3%, **4d**—89.7%) seemed to be the pair **3d**–**4d**, the oxazolone and triazinone that present both a bromine and a fluorine atom in their structures. In the presence of these compounds, the growth of the *S. cerevisiae* was practically unhindered (Figure 3). This was rather surprising, indicating that the increased tolerance to compounds **3a**/**4a**, **3b**/**4b** or **3d**/**4d** may be caused by active extrusion of the compounds from the cytosol via ATP-dependent multidrug transporters. As the apparent tolerance could be the result of pleiotropic drug resistance, the growth of yeast cells defective in plasma membrane multidrug transporters Pdr5 and Snq2 [41,42] exposed to various concentrations of compounds **3a**–**d** and **4a**–**d** (Figure 4) was determined.

It was noticed that both *pdr5Δ* and *snq2Δ* knockout mutants were more sensitive to compounds **3a**/**4a** and **3b**/**4b** and significantly more sensitive to **3d**/**4d** (Figure 4) than the wild type, suggesting that these compounds may be extruded from yeast cytosol by both Pdr5 and Snq2. In contrast, the sensitivity of yeast cells to **3c**/**4c** was not significantly altered by *PDR5* or *SNQ2* deletion (Figure 4). These observations suggest that either **3c**/**4c** are not recognized by Pdr5/Snq2, or the compounds act as inhibitors of Pdr5/Snq2 activity. Pdr5 and Snq2 play an important role in the efflux of xenobiotics, as their spectrum contains a wide variety of compounds, including anticancer drugs [42].

### 2.3. Prediction of the Molecular Mechanism of Action

The results of the PASS analysis for compounds were manually analyzed, and the relevant oncotargets are presented with the predicted Pa values in Table 2.

The Pa values are not an indicator of a compound’s potency but the probability that the compound will interact with a certain biological target or produce a specific effect. The Pa values indicate the inhibition of certain protein kinases as the major antiproliferative mechanism, especially the platelet-derived growth factor receptor kinase (PDGF-R) and focal adhesion kinase 2 (FAK2). The transformation of the oxazol-5(4*H*)-one fragment in the corresponding 1,2,4-triazin-6(5*H*)-one scaffold reduces the probability of FAK2 inhibition, but has little effect on the PDGF-R inhibitory potential.

**Figure 5:**
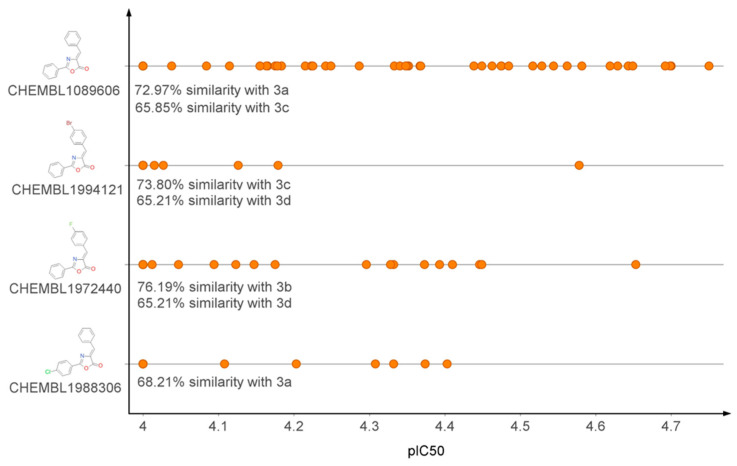
The antiproliferative profile on the NCI60 cell panel for the compounds with the best chemical similarities, with the compounds **3a**–**d** and **4a**–**d** achieving the highest.

The similarity search on the ChEMBL database returned a number of 61 structures for the compounds **3a**–**d** and only 2 results for the related **4a**–**d** structures. The highest degree of structural similarity (76.19%) was registered for the pair **3b** and CHEMBL1972440 (4-[(4-fluorophenyl)methylene]-2-phenyloxazol-5(4*H*)-one). The IC_50_ value represents the concentration expressed as a mol/L of each compound causing a 50% decrease in cell proliferation, and pIC_50_ represents the corresponding negative log10 value. Of the 61 similar compounds found, 20 were tested on the NCI60 cell assay. The pIC_50_ values for the 60 cells in the NCI database are graphically presented, with the best similarities being found for the compounds **3a**–**d** (Figure 5).

Four compounds, CHEMBL1972440, CHEMBL1994121, CHEMBL1089606 and CHEMBL1988306, share a common 4-(phenyl)methylene-2-phenyloxazol-5(4*H*)-one scaffold and average antiproliferative effects. The best anticancer profile was observed for CHEMBL1089606, indicating that the substitution on the aromatic rings with halogens is unfavorable. This result is similar to the results of the PASS prediction, which suggest compound **3a** has a better potential to inhibit protein kinases.

## 3. Materials and Methods

### 3.1. Chemistry

#### 3.1.1. General Information

All reagents and solvents were purchased commercially with high purity from Sigma–Aldrich or Merck. The melting points (m.p.) were determined using a Boëtius hot-plate microscope (VEB Wägetechnik Rapido, PHMK 81/3026, Radebeul, Germany) and are uncorrected. The IR spectra were registered on a Vertex 70 spectrometer (Bruker Optik GmbH, Ettlingen, Germany) in KBr pellets. The ^1^H-NMR (300 MHz) and ^13^C-NMR (75 MHz) spectra were recorded on a Gemini 300 BB spectrometer (Varian, Inc., Palo Alto, CA, USA) in deuterated DMSO-*d*_6_ solvent at room temperature. The values of chemical shifts (*δ*) in parts per million (ppm) are reported relative to tetramethylsilane (TMS) as the standard reference, and the coupling constants (*J*) are provided in Hz. The multiplicity of signals is abbreviated as follows: s, singlet; d, doublet; dd, doublet of doublets; t, triplet; m, multiplet; b, broad signal. The mass spectra of **3a**, **3d** and **4a**–**c** were registered on a triple-quadrupole Varian 1200 LC/MS/MS mass spectrometer (Varian, Inc. Walnut Creek, CA, USA) with an ESI (electrospray ionisation) or APCI (atmospheric pressure chemical ionization) interface. The mass spectrum for **4d** was acquired with the 8060NX triple-quadrupole mass spectrometer, with pump LC 40 D X3 automatic injector SIL 40 C X3 fitted with an ESI source (Shimadzu, Kyoto, Japan). Solutions of oxazolones **3** or triazinones **4** in chloroform (0.5 mg /mL) were prepared and diluted 10 times with methanol (1% formic acid for **3** and 1% ammonium carbonate for **4**). The sample solutions of compounds **3** were ionized positively, and those of **4** were ionized negatively by passing them through the ESI/APCI interface with positive or negative ionization using a solution injection system with a loop mounted on a Reodyne 7725 valve and the Varian Prostar 240 SDM pump, operating at a flow rate of 50 µL/min. The protonated molecular ions of oxazolones or negative ions of triazinones were fragmented into the argon collision cell at 1.5 mTorr. The elemental analysis was carried out on a Perkin-Elmer 2400 Series II CHNS/O Elemental Analyzer (Waltham, MA, USA).

#### 3.1.2. General Procedure for the Synthesis of 4-Arylidene-2-(4-(4-X-phenylsulfonyl)phenyl)oxazol-5(4*H*)-ones **3a**–**d**

A mixture of 2-(4-(4-X-phenylsulfonyl)benzamido)acetic acid **1** (10 mmol), aromatic aldehyde **2** (10 mmol), fused sodium acetate (10 mmol, 0.82 g) and acetic anhydride (19 mL) was added to a round-bottomed flask and was heated under reflux, with magnetic stirring, for 4 h. After the completion of the reaction time, ethanol (4 mL) was added, and the mixture left overnight at a cold temperature. The formed precipitate was filtered off, washed alternatively with boiling water and cold ethanol and then dried. The yellow product was recrystallized from an ethanol–chloroform mixture (1:2, *v*/*v*).

*4-Benzylidene-2-(4-(phenylsulfonyl)phenyl)oxazol-5(4H)-one* (**3a**), m.p. = 198–200 °C; Yield = 38%; FT-IR (KBr, *ν* cm^−1^): 3089 m, 3066 m, 3024 w, 3006 w, 1792 vs, 1771 vs, 1651 vs, 1595 m, 1552 m, 1323 vs, 1293 vs, 1160 vs, 1099 vs; ^1^H-NMR (DMSO-*d*_6_, *δ* ppm, *J* Hz): 7.43 (s, 1H, H-18), 7.51 (m, H-21, H-22, H-23), 7.65 (bd, 7.2, 2H, H-14, H-16), 7.74 (t, 7.2, 1H, H-15), 8.01 (bd, 7.3, 2H, H-13, H-17), 8.17 (d, 8.4, 2H, H-8, H-10), 8.23 (m, 2H, H-20, H-24), 8.25 (d, 8.4, 2H, H-7, H-11); ^13^C-NMR (DMSO-*d*_6_, *δ* ppm): 127.5 (C-13, C-17), 128.1 (C-8, C-10), 129.0 (C-21, C-23), 129.1 (C-7, C-11), 129.7 (C-6), 129.9 (C-14, C-16), 131.6 (C-22), 132.4 (C-20, C-24), 132.5 (C-18), 132.8 (C-19), 133.1 (C-4), 134.1 (C-15), 140.2 (C-12), 144.7 (C-9), 161.6 (C-2), 166.4 (C-5); Anal. (%): Calcd. for C_22_H_15_NO_4_S (389.42 g/mol): C, 67.85; H, 3.88; N, 3.60; S, 8.23. Found: C, 67.65; H, 3.90; N, 3.66; S, 8.30; +ESI-MS, *m*/*z* (%): 390 (73.9) [M + H]^+^, 245 (100, BP) [C_6_H_5_SO_2_C_6_H_4_CO]^+^, 125 (59.3) [C_6_H_5_SO]^+^.

*2-(4-(Phenylsulfonyl)phenyl)-4-(4-fluorobenzylidene)oxazol-5(4H)-one* (**3b**), m.p. = 241–243 °C; Yield = 80%; FT-IR (KBr, *ν* cm^−1^): 3101 w, 3064 w, 3048 w, 3003 w, 1798 vs, 1773 s, 1657 s, 1595 s, 1578 m, 1554 m, 1505 m, 1326 s, 1295 s, 1163 vs, 1097 s; ^1^H-NMR (DMSO-*d*_6_, *δ* ppm, *J* Hz): 7.36 (t, 8.7, 2H, H-21, H-23), 7.45 (s, 1H, H-18), 7.63 (bd, 7.4, 2H, H-14, H-16), 7.73 (t, 7.4, 1H, H-15), 8.02 (bd, 7.4, 2H, H-13, H-17), 8.18 (d, 8.2, 2H, H-8, H-10), 8.28 (d, 8.2, 2H, H-7, H-11), 8.36 (dd, 8.7, 6.0, 2H, H-20, H-24); ^13^C-NMR (DMSO-*d*_6_, *δ* ppm, *J* Hz): 116.6 (d, 21.9, C-21, C-23), 127.5 (C-13, C-17), 128.1 (C-8, C-10), 129.0 (C-7, C-11), 129.6 (C-6), 129.8 (C-14, C-16), 129.9 (C-19), 131.2 (C-18), 132.3 (C-4), 134.1 (C-15), 134.8 (d, 8.9, C-20, C-24), 140.1 (C-12), 144.7 (C-9), 161.5 (C-2), 163.6 (d, 285.7, C-22), 166.3 (C-5); Anal. (%): Calcd. for C_22_H_14_FNO_4_S (407.41 g/mol): C, 64.86; H, 3.46; N, 3.44; S, 7.87. Found: C, 64.74; H, 3.50; N, 3.35; S, 7.91.

*4-Benzylidene-2-(4-(4-bromophenylsulfonyl)phenyl)oxazol-5(4H)-one* (**3c**) [38], The ^13^C-NMR spectral data of the **3c** was not reported previously. ^13^C-NMR (DMSO-*d*_6_, *δ* ppm): 128.28 (C-13, C-17), 128.5 (C-15), 129.0 (C-7, C-11), 129.2 (C-21, C-23), 129.6 (C-8, C-10), 129.9 (C-6), 131.7 (C-22), 132.5 (C-20, C-24), 132.6 (C-18), 132.8 (C-19), 133.0 (C-14, C-16), 133.1 (C-4), 139.4 (C-12), 144.2 (C-9), 161.6 (C-2), 166.4 (C-5).

*2-(4-(4-Bromophenylsulfonyl)phenyl)-4-(4-fluorobenzylidene)oxazol-5(4H)-one* (**3d**), m.p. = 262–264 °C; Yield = 55%; FT-IR (KBr, *ν* cm^−1^): 3088 m, 3048 m, 3003 w, 1797 vs, 1773 vs, 1657 vs, 1596 vs, 1573 s, 1506 s, 1329 vs, 1292 s, 1161 vs, 1096 s, 611 s, 575 s; ^1^H-NMR (DMSO-*d*_6_, *δ* ppm, *J* Hz): 7.37 (t, 8.7, H-21, H-23), 7.46 (s, 1H, H-18), 7.87 (d, 8.8, 2H, H-14, H-16), 7.94 (d, 8.8, 2H, H-13, H-17), 8.20 (d, 8.2, 2H, H-8, H-10), 8.30 (d, 8.2, 2H, H-7, H-11), 8.38 (dd, 8.5, 5.9 Hz, 2H, H-20, H-24); ^13^C-NMR (DMSO-*d*_6_, *δ* ppm, *J* Hz): 117.0 (d, 21.9, C-21, C-23), 128.3 (C-13, C-17), 128.7 (C-15), 129.2 (C-14, C-16), 129.3 (C-7, C-11), 129.6 (C-8, C-10), 129.6 (C-6), 129.9 (C-19), 131.3 (C-18), 135.0 (C-4), 135.1 (d, 10.1, C-20, C-24), 139.4 (C-12), 144.2 (C-9), 161.7 (C-2), 163.3 (d, 286.2, C-22), 166.4 (C-5); Anal. (%): Calcd. for C_22_H_13_BrFNO_4_S (486.31 g/mol): C, 54.33; H, 2.69; N, 2.88; S, 6.59. Found: C, 54.14; H, 2.75; N, 2.89; S, 6.81; +APCI-MS, *m*/*z* (%): 486 (7.6) [^79^Br M + H]^+^, 488 (8.1) [^81^Br M + H]^+^, 323 (100, BP) [^79^BrC_6_H_4_SO_2_C_6_H_4_CO]^+^, 325 (100, BP) [^81^BrC_6_H_4_SO_2_C_6_H_4_CO]^+^, 203 (38.2) [^79^BrC_6_H_4_SO]^+^, 205 (20.1) [^81^BrC_6_H_4_SO]^+^.

#### 3.1.3. General Procedure for the Synthesis of 3-(4-(4-X-Phenylsulfonyl)phenyl)-5-(4-arylidene)-2-phenyl-1,2-dihydro-1,2,4-triazin-6(5*H*)-ones **4a**–**d**

To a solution of oxazolone **3** (3 mmol) in acetic acid (7.5 mL), phenylhydrazine (3 mmol) and fused sodium acetate (0.45 mmol, 36.9 mg) were added. The mixture was heated under reflux, with magnetic stirring for 5 h. The obtained precipitate was cooled, filtered and washed with hot water, and then dried, and the yellow solid was recrystallized from ethanol.

*5-Benzylidene-3-(4-(phenylsulfonyl)phenyl)-2-phenyl-1,2-dihydro-1,2,4-triazin-6(5H)-one* (**4a**), m.p. = 253–254 °C; Yield = 42%; FT-IR (KBr, *ν* cm^−1^): 3231 w, 3065 w, 3032 w, 1715 vs, 1640 m, 1595 w, 1494 w, 1323 m, 1290 s, 1157 vs; ^1^H-NMR (DMSO-*d*_6_, *δ* ppm, *J* Hz): 6.73 (d, 8.2, 2H, H-26, H-30), 6.82 (t, 7.3, 1H, H-28), 7.19 (t, 7.2, 2H, H-27, H-29), 7.33 (s, 1H, H-18), 7.50 (m, 2H, H-22, H-23), 7.52 (m, 1H, H-21), 7.62 (t, 7.7, 2H, H-14, H-16), 7.70 (t, 7.7, 1H, H-15), 7.99 (d, 7.5, 2H, H-13, H-17), 8.11 (d, 8.6, 2H, H-8, H-10), 8.31 (d, 8.6, 2H, H-7, H-11), 8.37 (m, H-20, H-24), 8.98 (s, 1H, NH); ^13^C-NMR (DMSO-*d*_6_, *δ* ppm): 112.5 (C-26, C-30), 120.4 (C-28), 127.6 (C-13, C-17), 127.7 (C-8, C-10), 128.9 (C-21, C-23), 129.3 (C-27, C-29), 129.7 (C-7, C-11), 129.8 (C-14, C-16), 129.9 (C-18), 131.1 (C-22), 132.2 (C-6), 132.8 (C-20, C-24), 133.7 (C-5), 134.1 (C-15), 136.1 (C-19), 140.4 (C-12), 143.7 (C-9), 146.2 (C-25), 159.6 (C-3), 168.8 (C=O); Anal. (%): Calcd. for C_28_H_21_N_3_O_3_S (479.55 g/mol): C, 70.13; H, 4.41; N, 8.76; S, 6.69. Found: C, 69.99; H, 4.46; N, 8.89; S, 6.80;−APCI-MS, *m*/*z* (%): 478 (35.8), [M − H]^−^, 387 (100, BP) [M − H − C_6_H_5_N]^−^, 116 (24.3) [C_6_H_5_CCNH]^−^.

*2-Phenyl-3-(4-(phenylsulfonyl)phenyl)-5-(4-fluorobenzylidene)-1,2-dihydro-1,2,4-triazin-6(5H)-one* (**4b**), m.p. = 262–264 °C; Yield = 83%; FT-IR (KBr, *ν* cm^−1^): 3312 m, 3098 w, 3067 w, 3041 w, 1709 vs, 1643 m, 1597 s, 1506 s, 1309 s, 1288 s, 1158 vs, 1105 m; ^1^H-NMR (DMSO-*d*_6_, *δ* ppm, *J* Hz): 6.73 (d, 7.5, 2H, H-26, H-30), 6.82 (t, 7.5, 1H, H-28), 7.19 (t, 7.5, 2H, H-27, H-29), 7.32 (t, 8.5, 2H, H-21, H-23), 7.35 (s, 1H, H-18), 7.62 (t, 7.2, 2H, H-14, H-16), 7.70 (t, 7.2, 1H, H-15), 7.98 (d, 7.2, 2H, H-13, H-17), 8.10 (d, 8.5, 2H, H-8, H-10), 8.31 (d, 8.5, 2H, H-7, H-11), 8.44 (dd, 8.5, 6.0, H-20, H-24), 8.98 (s, 1H, NH); ^13^C-NMR (DMSO-*d*_6_, *δ* ppm, *J* Hz): 112.5 (C-26, C-30), 116.1 (d, 21.6, C-21, C-23), 120.4 (C-28), 127.6 (C-8, C-10), 128.5 (C-18), 129.3 (C-14, C-16, C-27, C-29), 129.8 (C-13, C-17), 129.8 (C-7, C-11), 130.4 (C-19), 132.1 (C-6), 134.1 (C-15), 135.3 (d, 8.8 Hz, C-20, C-24), 135.7 (C-5), 140.3 (C-12), 143.8 (C-9), 146.1 (C-25), 159.6 (C-3), 164.4 (d, 251.3, C-22), 168.7 (C=O); Anal. (%): Calcd. for C_28_H_20_FN_3_O_3_S (497.54 g/mol): C, 67.59; H, 4.05; N, 8.45; S, 6.44. Found: C, 67.54; H, 4.15; N, 8.29; S, 6.49; −APCI-MS, *m*/*z* (%): 496 (12.1) [M − H]^−^, 405 (100, BP) [M − H − C_6_H_5_N]^−^, 134 (60.6) [FC_6_H_4_CCNH]^−^.

*5-Benzylidene-3-(4-(4-bromophenylsulfonyl)phenyl)-2-phenyl-1,2-dihydro-1,2,4-triazin-6(5H)-one* (**4c**), m.p. = 267–269 °C; Yield = 84%; FT-IR (KBr, *ν* cm^−1^): 3331 m, 3087 w, 3067 m, 3040 w, 1717 vs, 1641 s, 1597 s, 1573 s, 1326 vs, 1294 vs, 1155 vs, 1101 vs, 615 s, 573 m; ^1^H-NMR (DMSO-*d*_6_, *δ* ppm, *J* Hz): 6.73 (t, 7.7, 1H, H-28), 6.74 (d, 7.7, 2H, H-26, H-30), 7.19 (t, 7.5, 2H, H-27, H-29), 7.34 (s, 1H, H-18), 7.35 (d, 8.1, 2H, H-13, H-17), 7.50 (m, 3H, H-21, H-22, H-23), 7.81 (d, 8.1, 2H, H-14, H-16), 8.11 (d, 8.2, 2H, H-8, H-10), 8.31 (d, 8.2, 2H, H-7, H-11), 8.33 (m, H-20, H-24), 9.00 (s, 1H, NH); ^13^C-NMR (DMSO-*d*_6_, *δ* ppm): 112.5 (C-26, C-30), 120.4 (C-28), 127.7 (C-8, C-10), 128.3 (C-15), 128.9 (C-13, C-17), 129.3 (C-21, C-23), 129.5 (C-18), 129.6 (C-27, C-29), 129.8 (C-7, C-11), 131.1 (C-22), 132.3 (C-6), 132.8 (C-14, C-16), 132.9 (C-20, C-24), 133.7 (C-5), 136.1 (C-19), 139.5 (C-12), 143.2 (C-9), 146.2 (C-25), 159.5 (C-3), 168.8 (C=O); Anal. (%): Calcd. for C_28_H_20_BrN_3_O_3_S (558.45 g/mol): C, 60.22; H, 3.61; N, 7.52; S, 5.74. Found: C, 59.97; H, 3.56; N, 7.41; S, 5.92; −APCI-MS, *m*/*z* (%): 556 (92.2) [^79^Br M − H]^−^, 558 (100, BP) [^81^Br M − H]^−^, 465 (100, BP) [^79^Br M − H − C_6_H_5_N]^−^, 467 (80.1) [^81^Br M − H − C_6_H_5_N]^−^.

*3-(4-(4-Bromophenylsulfonyl)phenyl)-5-(4-fluorobenzylidene)-2-phenyl-1,2-dihydro-1,2,4-triazin-6(5H)-one* (**4d**), m.p. = 267–269 °C; Yield = 76%; FT-IR (KBr, *ν* cm^−1^): 3330 m, 3093 w, 3075 w, 3039 w, 1720 vs, 1642 s, 1596 vs, 1574 s, 1505 vs, 1327 vs, 1297 vs, 1156 vs, 1101 vs, 613 vs, 573 s; ^1^H-NMR (DMSO-*d*_6_, *δ* ppm, *J* Hz): 6.72 (d, 7.4, 2H, H-26, H-30), 6.82 (t, 7.4, 1H, H-28), 7.19 (t, 7.4, 2H, H-27, H-29), 7.31 (t, 8.5, 2H, H-21, H-23), 7.36 (s, 1H, H-18), 7.83 (d, 8.8, 2H, H-14, H-16), 7.91 (d, 8.8, 2H, H-13, H-17), 8.11 (d, 8.5, 2H, H-8, H-10), 8.32 (d, 8.5, 2H, H-7, H-11), 8.45 (dd, 8.7, 5.8, H-20, H-24), 8.99 (s, 1H, NH); ^13^C-NMR (DMSO-*d*_6_, *δ* ppm, *J* Hz): 112.5 (C-26, C-30), 116.2 (d, 21.7, C-21, C-23), 120.4 (C-28), 127.7 (C-8, C-10), 128.4 (C-15), 128.6 (C-18), 129.4 (C-13, C-17), 129.6 (C-27, C-29), 129.9 (C-7, C-11), 132.2 (C-19), 132.3 (C-6), 133.0 (C-14, C-16), 135.4 (d, 8.5, C-20, C-24), 135.70 (C-5), 139.6 (C-12), 143.2 (C-9), 146.2 (C-25), 159.7 (C-3), 163.5 (d, 263.8, C-22), 168.8 (C=O); Anal. (%): Calcd. for C_28_H_19_BrFN_3_O_3_S (576.44 g/mol): C, 58.34; H, 3.32; N, 7.29; S, 5.56. Found: C, 58.27; H, 3.42; N, 7.09; S, 5.67; −ESI-MS, *m*/*z* (%): 574 (100, BP) [^79^Br M − H]^−^, 576 (100, BP) [^81^Br M − H]^−^, 483 (66.2) [^79^Br M − H − C_6_H_5_N]^−^, 485 (93.8) [^81^Br M − H − C_6_H_5_N]^−^.

### 3.2. Toxicity Evaluation

#### 3.2.1. *Daphnia magna* Toxicity Assay

*D. magna* Straus was cultured parthenogenetically at 25 °C, with a 16 h/8 h light–dark cycle. Young daphnids were selected based on their size and kept in an artificial medium for 24 h prior to the bioassay. The determination was conducted in tissue culture plates containing 12 wells (Greiner Bio-One, Kremsmünster, Austria), with 10 organisms in each well at a final volume of 4 mL/sample [48,49]. Dimethyl sulfoxide of 1% concentration was used as the negative control. Compounds **3a**–**d** and **4a**–**d** were tested at six concentration levels ranging from 8 to 198 μg/mL. All determinations were performed in duplicate. Lethality was observed at 24 and 48 h, and the LC_50_ values were calculated for each compound using the least-square fit method. The LC_50_ and 95% confidence interval of LC_50_ ( 95% CI) were also calculated with the same method using GraphPad Prism v 5.1 software.

#### 3.2.2. *S. cerevisiae* Toxicity Assay

##### Yeast Strain and Growth Conditions

The *S. cerevisiae* strains used in this study were isogenic to of BY4741 *(MATa; his3Δ1; leu2Δ0; met15Δ0; ura3Δ0)* [50], considered the wild-type (WT) strain. The single-gene deletion (knockout) strains used were Y02409 (BY4741, *pdr5::kanMX4*, denoted *pdr5Δ*) and Y03951 (BY4741, *snq2::kanMX4*, denoted *snq2Δ*) [51]. The strains were purchased from EUROSCARF, Frankfurt, Germany (www.euroscarf.de, accesesd on 1 May 2023). Cell storage, growth and manipulation were carried out as described. The strains were grown in rich YPD medium (1% *w*/*v* yeast extract, 2% *w*/*v* peptone, 2% *w*/*v* glucose) or in synthetic complete medium (SC—0.67% *w*/*v* yeast nitrogen base with (NH_4_)_2_SO_4_, 2% *w*/*v* glucose, supplemented with the necessary amino acids) [52]. After autoclaving and cooling to 60 °C, sterile solutions of the tested compounds (20 mM in dimethyl sulfoxide stock solutions) were added to the yeast media.

##### Cell Growth Assessment

Yeast pre-cultures left overnight in rich YPD medium were inoculated in fresh SC medium at a density of 2 × 10^5^ cells/mL, and incubated afterwards for 2 h under shaking (200 rpm, 30 °C) in a multi-amplitude orbital constant temperature shaking incubator (Shanghai ZHICHENG Analytical Instruments Manufacturing Co., Ltd., Shanghai, China) before the solutions of the compounds **3a**–**d** and **4a**–**d** were added to the specified concentrations.

The growth of the yeast was determined after 24 h of exposure to the oxazolones and triazinones by measuring the turbidity of the cellular suspensions at the wavelength of 600 nm [53]. The turbidity was recorded using a plate reader equipped with a thermostat and a shaker (Varioskan, Thermo Fisher Scientific, Vantaa, Finland). The growth of the yeast cells in the presence of each tested compound was calculated relative to the cell growth in the absence of the compound, in a medium containing the equivalent concentration of dimethyl sulfoxide.

### 3.3. Prediction of the Molecular Mechanism of Action

The SMILES codes for the compounds **3a**–**d** and **4a**–**d** were introduced in the PASS (Prediction of Activity Spectra for Substances) application in order to evaluate the potential to interact with a large collection of biological relevant molecules. The output results consist of an array of pair probabilities, Pa and Pi. Pa represents the probability of the compound to interact (Pa) with a specific target, while Pi is the probability of the negative outcome (Pi) [54].

For each compound **3a**–**d** and **4a**–**d**, a similarity search was performed on the ChEMBL database using a 50% threshold [49,55]. The output structures were extracted together with their antiproliferative data on the NCI cell panel. The collected data were filtered using DataWarrior v5.2.1 software [56].

## 4. Conclusions

New compounds from oxazol-5(4*H*)-one and 1,2,4-triazin-6(5*H*)-one classes incorporating a diaryl sulfone moiety were synthesized and characterized, and their toxicity was evaluated on *D. magna* crustaceans and *S. cerevisiae* yeast. The new unsaturated oxazolones were prepared via cyclocondensation of some 2-(4-(4-X-phenylsulfonyl)benzamido)acetic acid intermediates with aromatic aldehydes. The new triazinone derivatives were synthesized from their oxazolone precursors via condensation with phenylhydrazine. The structures of the new synthesized compounds were established using IR, ^1^H-, ^13^C-NMR, mass spectral data and elemental analysis. Both the heterocyclic nucleus and the halogen atoms significantly influenced the toxicity against *D. magna*. The oxazolone derivatives were less toxic compared with the compounds belonging to triazinone series. The results of toxicity screening against *S. cerevisiae* indicate that the action of the compounds is considerably hindered by the activity of the MDR transporters Pdr5 and Snq2. The predictive studies indicated that the new compounds could inhibit cancer cells proliferation by targeting certain protein kinases, especially PDGF-R and FAK2. The results suggest that compound **3a** has the best potential to inhibit oncologic protein kinases, and an average antiproliferative effect with pIC_50_ values between 4 and 5. The same derivative had the lowest toxicity in the *D. magna* assay, thus being a good candidate for future anticancer investigations. All the results obtained in the investigation of the synthesized compounds require further study for the elucidation of the mechanisms implicated.

## Figures and Tables

**Table 1:** Results of *Daphnia magna* bioassay.

Compound	LC_50 48h_ ^1^ (µM)	95% CI_48h_ ^2^ (µM)
**3a**	482.6	264.6–880.3
**3b**	319	268.5–378.9
**3c**	195.2	131.6–289.7
**3d**	227.1	143.2–360.1
**4a**	65.53	45.47–94.44
**4b**	34.68	30.67–39.22
**4c**	46.06	30.13–70.42
**4d**	102.5	86.06–122.1

^1^ LC_50_—50% lethal concentration; ^2^ 95% CI—95% confidence interval.

**Table 2:** The probability (Pa) that compounds **3a**–**d** and **4a**–**d** will be active towards oncotargets as predicted by PASS analysis.

Effect	Compound							
	3a	3b	3c	3d	4a	4b	4c	4d
Angiogenesis inhibitor	0.53	0.48	0.52	0.48	-	-	-	-
Antineoplastic (all)	-	-	-	-	0.37	0.31	0.37	0.31
Antineoplastic (lung cancer)	-	-	-	-	0.25	0.19	0.21	0.17
Antineoplastic (pancreatic cancer)	-	-	0.22	0.21	-	-	-	-
Antineoplastic (sarcoma)	-	-	0.17	0.16				
Kinase inhibitor	0.44	0.33	0.33	-	0.38	0.30	0.30	-
Aurora-C kinase inhibitor	-	-	0.21	0.20	0.11	0.11	0.23	0.22
Focal adhesion kinase 2 inhibitor	0.39	0.36	0.32	0.30	0.24	0.21	-	-
Focal adhesion kinase inhibitor	0.27	0.26	0.23	0.22	-	-	-	-
Platelet-derived growth factor receptor kinase inhibitor	0.37	0.43	0.27	0.33	0.43	0.47	0.34	0.39
Protein kinase CK1 inhibitor	-	-		0.13		0.14	0.14	0.18

## Data Availability

The datasets used and/or analyzed during the current study are available from the corresponding author on reasonable request.

## Supplementary Materials

The following supporting information can be downloaded at https://www.mdpi.com/article/10.3390/molecules28124834/s1.

## References

[1] Jampilek J. (2019). Heterocycles in Medicinal Chemistry. Molecules.

[2] Heravi M.M., Zadsirjan V. (2020). Prescribed Drugs Containing Nitrogen Heterocycles: An Overview. RSC Adv..

[3] Pozharskii A.F., Soldatenkov A.T., Katritzky A.R. (2011). Heterocycles in Life and Society: An Introduction to Heterocyclic Chemistry, Biochemistry and Applications.

[4] Kakkar S., Narasimhan B. (2019). A Comprehensive Review on Biological Activities of Oxazole Derivatives. BMC Chem..

[5] Wei L., Yuan G. (2023). Synthesis of 2,4,5-Trisubstituted Oxazoles from 1,2-Diketones and Amines by Using an Electrochemical Method. Tetrahedron.

[6] Samuel Y., Garg A., Mulugeta E. (2021). Synthesis, DFT Analysis, and Evaluation of Antibacterial and Antioxidant Activities of Sulfathiazole Derivatives Combined with In Silico Molecular Docking and ADMET Predictions. Biochem. Res. Int..

[7] Kushwaha N., Kushwaha S. (2022). Synthetic Approaches and Biological Significance of Oxazolone Moieties: A Review. Biointerface Res. Appl. Chem..

[8] Youssef A.S.A., El-Mariah F.A., Abd-Elmottaleb F.T., Hashem H.E. (2019). Reaction of 2-Phenyl-4-Arylidene-1,3-Oxazolones with Different Nucleophiles for Synthesis of Some New Heterocycles. J. Heterocycl. Chem..

[9] Al-Warhi T., Abualnaja M., Abu Ali O.A., Althobaiti F., Alharthi F., Elsaid F.G., Shati A.A., Fayad E., Elghareeb D., Abu Almaaty A.H. (2022). Synthesis and Biological Activity Screening of Newly Synthesized Trimethoxyphenyl-Based Analogues as Potential Anticancer Agents. Molecules.

[10] Mavridis E., Bermperoglou E., Pontiki E., Hadjipavlou-Litina D. (2020). 5-(4*H*)-Oxazolones and Their Benzamides as Potential Bioactive Small Molecules. Molecules.

[11] Yu C., Zhou B., Su W., Xu Z. (2006). Erlenmeyer Synthesis for Azlactones Catalyzed by Ytterbium(III) Triflate under Solvent-Free Conditions. Synth. Commun..

[12] Algohary A.M., Alhalafi M.H. (2022). Design, Synthesis and Evaluate of Imidazole, Triazine and Metastable Oxazolone Derivatives as Chemosensor for Detecting Metals. J. Saudi Chem. Soc..

[13] Almalki A.J., Ibrahim T.S., Taher E.S., Mohamed M.F.A., Youns M., Hegazy W.A.H., Al-Mahmoudy A.M.M. (2022). Synthesis, Antimicrobial, Anti-Virulence and Anticancer Evaluation of New 5(4*H*)-Oxazolone-Based Sulfonamides. Molecules.

[14] Savariz F.C., Foglio M.A., De Carvalho J.E., Ruiz A.L.T.G., Duarte M.C.T., Da Rosa M.F., Meyer E., Sarragiotto M.H. (2012). Synthesis and Evaluation of New β-Carboline-3-(4-Benzylidene)-4*H*-Oxazol-5-One Derivatives as Antitumor Agents. Molecules.

[15] Albelwi F.F., Al-anazi M., Naqvi A., Hritani Z.M., Okasha R.M., Afifi T.H., Hagar M. (2021). Novel Oxazolones Incorporated Azo Dye: Design, Synthesis Photophysical-DFT Aspects and Antimicrobial Assessments with In-Silico and In-Vitro Surveys. J. Photochem. Photobiol..

[16] Parveen M., Ali A., Ahmed S., Malla A.M., Alam M., Pereira Silva P.S., Silva M.R., Lee D.U. (2013). Synthesis, Bioassay, Crystal Structure and Ab Initio Studies of Erlenmeyer Azlactones. Spectrochim. Acta A Mol. Biomol. Spectrosc..

[17] Saour K.Y., Al-Bayati R.I.H., Shia J.S. (2015). Synthesis of 4-Benzylidene-2-(4-Nitro-Phenyl)-4*H*-Oxazol-5-One Derivatives with Suspected Biological Activity. Chem. Mat. Res..

[18] Mohamed L.W., El-Badry O.M., El-Ansary A.K., Ismael A. (2017). Design & Synthesis of Novel Oxazolone & Triazinone Derivatives and Their Biological Evaluation as COX-2 Inhibitors. Bioorg. Chem..

[19] Sun Y.-X., Xu G.-L., Hou B.-B., Ao G.-Z. (2015). Synthesis and Anti-Inflammatory Activities of Oxazolone Compounds. Chin. J. Synth. Chem..

[20] Kuş C., Uğurlu E., Özdamar E.D., Can-Eke B. (2017). Synthesis and Antioxidant Properties of New Oxazole-5(4*H*)-One Derivatives. Turk. J. Pharm. Sci..

[21] Mariappan G., Saha B.P., Datta S., Kumar D., Haldar P.K. (2011). Design, Synthesis and Antidiabetic Evaluation of Oxazolone Derivatives. J. Chem. Sci..

[22] Kumar R., Kumar N., Roy R.K., Singh A. (2017). Triazines—A Comprehensive Review of Their Synthesis and Diverse Biological Importance. Curr. Med. Drug Res..

[23] Cascioferro S., Parrino B., Spanò V., Carbone A., Montalbano A., Barraja P., Diana P., Cirrincione G. (2017). An Overview on the Recent Developments of 1,2,4-Triazine Derivatives as Anticancer Compounds. Eur. J. Med. Chem..

[24] Pal R., Kumar B., Guruubasavaraja S.P.M., Chawla P.A. (2023). Design, Synthesis of 1,2,4-Triazine Derivatives as Antidepressant and Antioxidant Agents: In Vitro, in Vivo and in Silico Studies. Bioorg. Chem..

[25] El-Barbary A.A., Imam D.R., El–Tahawy M.M.T., El-Hallouty S.M., Kheder N.A., Khodair A.I. (2023). Unexpected Synthesis, Characterization, Biological Evaluations, and Computational Details of Novel Nucleosides Containing Triazine-Pyrrole Hybrid. J. Mol. Struct..

[26] El-Megharbel S.M., Alaryani F.S., Qahl S.H., Hamza R.Z. (2022). Synthesis, Spectroscopic Studies for Five New Mg (II), Fe (III), Cu (II), Zn (II) and Se (IV) Ceftriaxone Antibiotic Drug Complexes and Their Possible Hepatoprotective and Antioxidant Capacities. Antibiotics.

[27] Zaki I., Abdelhameid M.K., El-Deen I.M., Abdel Wahab A.H.A., Ashmawy A.M., Mohamed K.O. (2018). Design, Synthesis and Screening of 1, 2, 4-Triazinone Derivatives as Potential Antitumor Agents with Apoptosis Inducing Activity on MCF-7 Breast Cancer Cell Line. Eur. J. Med. Chem..

[28] Salem M.S., El-Helw E.A.E., Derbala H.A.Y. (2020). Development of Chromone–Pyrazole-Based Anticancer Agents. Russ. J. Bioorg. Chem..

[29] Verma T., Sinha M., Bansal N. (2019). Triazinone Derivatives as Antibacterial and Antimalarial Agents. Asian Pac. J. Health Sci..

[30] Kaushik D., Khan S.A., Chawla G. (2010). Design & Synthesis of 2-(Substituted Aryloxy)-5-(Substituted Benzylidene)-3-Phenyl-2,5-Dihydro-1H-[1,2,4] Triazin-6-One as Potential Anticonvulsant Agents. Eur. J. Med. Chem..

[31] Al-Said M.S., Ghorab M.M., Nissan Y.M. (2012). Dapson in Heterocyclic Chemistry, Part VIII: Synthesis, Molecular Docking and Anticancer Activity of Some Novel Sulfonylbiscompounds Carrying Biologically Active 1,3-Dihydropyridine, Chromene and Chromenopyridine Moieties. Chem. Cent. J..

[32] Nematollahi D., Khazalpour S., Ranjbar M., Momeni S. (2017). A Green Strategy for the Synthesis of Sulfone Derivatives of *p*-Methylaminophenol: Kinetic Evaluation and Antibacterial Susceptibility. Sci. Rep..

[33] Mady M.F., Awad G.E.A., Jørgensen K.B. (2014). Ultrasound-Assisted Synthesis of Novel 1,2,3-Triazoles Coupled Diaryl Sulfone Moieties by the CuAAC Reaction, and Biological Evaluation of Them as Antioxidant and Antimicrobial Agents. Eur. J. Med. Chem..

[34] Neamati N., Mazumder A., Zhao H., Sunder S., Burke T.R., Schultz R.J., Pommier Y. (1997). Diarylsulfones, a Novel Class of Human Immunodeficiency Virus Type 1 Integrase Inhibitors. Antimicrob. Agents Chemother..

[35] Ghaoui N., Hanna E., Abbas O., Kibbi A.G., Kurban M. (2020). Update on the Use of Dapsone in Dermatology. Int. J. Dermatol..

[36] Roșca E.V., Apostol T.V., Chifiriuc M.C., Grădișteanu Pîrcălăbioru G., Drăghici C., Socea L.I., Olaru O.T., Nițulescu G.M., Pahonțu E.M., Hrubaru M. (2020). In Silico and Experimental Studies for the Development of Novel Oxazol-5(4*H*)-Ones with Pharmacological Potential. Farmacia.

[37] Rosca E.V., Apostol T.V., Draghici C., Olaru O.T., Socea L.I., Iscrulescu L., Saramet G., Barbuceanu F., Pahontu E.M., Baraitareanu S. (2019). Synthesis, Characterization and Cytotoxicity Evaluation of New Compounds from Oxazol-5(4*H*)-Ones and 1,2,4-Triazin-6(5*H*)-Ones Classes. Rev. Chim..

[38] Bărbuceanu F., Roşca E.V., Apostol T.V., Şeremet O.C., Drăghici C., Mihai D.P., Negreş S., Niţulescu G.M., Bărbuceanu Ş.F. (2020). New 2-(4-(4-Bromophenylsulfonyl)Phenyl)-4-Arylidene-Oxazol-5(4*H*)-Ones: Analgesic Activity and Histopathological Assessment. Rom. J. Morphol. Embryol..

[39] Apostol T.V., Marutescu L.G., Draghici C., Socea L.I., Olaru O.T., Nitulescu G.M., Pahontu E.M., Saramet G., Enache-Preoteasa C., Barbuceanu S.F. (2021). Synthesis and Biological Evaluation of New *N*-Acyl-α-amino Ketones and 1,3-Oxazoles Derivatives. Molecules.

[40] Guilhermino L., Diamantino T., Carolina Silva M., Soares A.M.V.M. (2000). Acute Toxicity Test with *Daphnia magna*: An Alternative to Mammals in the Prescreening of Chemical Toxicity?. Ecotoxicol. Environ. Saf..

[41] Schmitt M., Schwanewilm P., Ludwig J., Lichtenberg-Fraté H. (2006). Use of PMA1 as a Housekeeping Biomarker for Assessment of Toxicant-Induced Stress in *Saccharomyces cerevisiae*. Appl. Environ. Microbiol..

[42] Kolaczkowski M., Goffeau A. (1997). Active Efflux by Multidrug Transporters as One of the Strategies to Evade Chemotherapy and Novel Practical Implications of Yeast Pleiotropic Drug Resistance. Pharmacol. Ther..

[43] Haneen D.S.A., Abou-Elmagd W.S.I., Youssef A.S.A. (2021). 5(4*H*)-Oxazolones: Synthesis and Biological Activities. Synth. Commun..

[44] Schiketanz I., Draghici C., Saramet I., Balaban A.T. (2002). Aminoketone, Oxazole and Thiazole Synthesis. Part 15.^1^ 2-[4-(4-Halobenzenesulphonyl)-Phenyl]-5-Aryloxazoles. Arkivoc.

[45] Schiketanz I., Draghici C., Saramet I., Balaban A.T. (2002). Aminoketone, Oxazole and Thiazole Synthesis. Part 16.^1^ Novel 5-aryl-2-(*p*ara-Benzenesulfonylphenyl)-Oxazoles. Rev. Roum. Chim..

[46] Apostol T.-V., Barbuceanu S.-F., Olaru O.T., Draghici C., Saramet G., Socea B., Enache C., Socea L.-I. (2019). Synthesis, Characterization and Cytotoxicity Evaluation of New Compounds from Oxazol-5(4*H*)-Ones and Oxazoles Class Containing 4-(4-Bromophenylsulfonyl)Phenyl Moiety. Rev. Chim..

[47] Rodrigues C.A.B., Mariz I.F.A., Maçôas E.M.S., Afonso C.A.M., Martinho J.M.G. (2013). Unsaturated Oxazolones as Nonlinear Fluorophores. Dyes. Pigm..

[48] Nitulescu G., Nicorescu I.M., Olaru O.T., Ungurianu A., Mihai D.P., Zanfirescu A., Nitulescu G.M., Margina D. (2017). Molecular Docking and Screening Studies of New Natural Sortase A Inhibitors. Int. J. Mol. Sci..

[49] Stecoza C.E., Nitulescu G.M., Draghici C., Caproiu M.T., Olaru O.T., Bostan M., Mihaila M. (2021). Synthesis and Anticancer Evaluation of New 1,3,4-Oxadiazole Derivatives. Pharmaceuticals.

[50] Brachmann C.B., Davies A., Cost G.J., Caputo E., Li J., Hieter P., Boeke J.D. (1998). Designer Deletion Strains Derived from *Saccharomyces cerevisiae* S288C: A Useful Set of Strains and Plasmids for PCR-Mediated Gene Disruption and Other Applications. Yeast.

[51] Kelly D.E., Lamb D.C., Kelly S.L. (2001). Genome-Wide Generation of Yeast Gene Deletion Strains. Comp. Funct. Genom..

[52] Sherman F. (2002). Getting Started with Yeast. Methods Enzymol..

[53] Amberg D.C., Burke D., Strathern J.N., Burke D., Dawson D., Stearns T. (2005). Measuring Yeast Cell Density by Spectrophotometry. Methods in Yeast Genetics: A Cold Spring Harbor Laboratory Course Manual.

[54] Nitulescu G.M., Iancu G., Nitulescu G., Iancu R.C., Bogdanici C., Vasile D. (2017). Brave New Hope for Breast Cancer Aminopyrazole Derivates between Rational Design and Clinical Efficacy. Rev. Chim..

[55] Mendez D., Gaulton A., Bento A.P., Chambers J., De Veij M., Félix E., Magariños M.P., Mosquera J.F., Mutowo P., Nowotka M. (2019). ChEMBL: Towards Direct Deposition of Bioassay Data. Nucleic Acids Res..

[56] Sander T., Freyss J., von Korff M., Rufener C. (2015). DataWarrior: An Open-Source Program For Chemistry Aware Data Visualization And Analysis. J. Chem. Inf. Model..

