# Supplementary material for: New Heterocyclic Compounds from Oxazol-5(4H)-one and 1,2,4-Triazin-6(5H)-one Classes: Synthesis, Characterization and Toxicity Evaluation
Source: Molecules. 2023 Jun 17;28(12):4834. doi: 10.3390/molecules28124834 (PMC10302834; doi:10.3390/molecules28124834)
Supplement: Supplementary file 1 [file molecules-28-04834-s001.zip › molecules-2443763-supplementary.pdf]

Supplementary Material

New Heterocyclic Compounds from Oxazol-5(4*H*)-one and 1,2,4-Triazin-6(5*H*)-one Classes: Synthesis, Characterization and Toxicity Evaluation

Stefania-Felicia Barbuceanu [1,*], Elena-Valentina Rosca [1], Theodora-Venera Apostol [1,*], Laura-Ileana Socea [1], Constantin Draghici [2], Ileana Cornelia Farcasanu [3], Lavinia Liliana Ruta [3], George Mihai Nitulescu [4], Lucian Iscrulescu [1], Elena-Mihaela Pahontu [5], Rica Boscencu [5], Gabriel Saramet [6] and Octavian Tudorel Olaru [7]

[1] Department of Organic Chemistry, Faculty of Pharmacy, "Carol Davila" University of Medicine and Pharmacy, 6 Traian Vuia Street, 020956 Bucharest, Romania; elenavalentinarosca90@gmail.com (E.-V.R.); laura.socea@umfcd.ro (L.-I.S.); lucian.iscrulescu@umfcd.ro (L.I.)

[2] "C. D. Nenitescu" Institute of Organic and Supramolecular Chemistry Romanian Academy, 202B Splaiul Independenței, 060023 Bucharest, Romania; cst.drag@yahoo.com (C.D.)

[3] Department of Organic Chemistry, Biochemistry and Catalysis, Faculty of Chemistry, University of Bucharest, 90–92 Panduri Str., 050663 Bucharest, Romania; ileana.farcasanu@chimie.unibuc.ro (I.C.F.); lavinia.ruta@chimie.unibuc.ro (L.L.R.)

[4] Department of Pharmaceutical Chemistry, Faculty of Pharmacy, "Carol Davila" University of Medicine and Pharmacy, 6 Traian Vuia Street, 020956 Bucharest, Romania; george.nitulescu@umfcd.ro (G.M.N.)

[5] Department of General and Inorganic Chemistry, Faculty of Pharmacy, "Carol Davila" University of Medicine and Pharmacy, 6 Traian Vuia Street, 020956 Bucharest, Romania; elena.pahontu@umfcd.ro (E.-M.P.); rica.boscencu@umfcd.ro (R.B.)

[6] Department of Pharmaceutical Technology and Biopharmacy, Faculty of Pharmacy, "Carol Davila" University of Medicine and Pharmacy, 6 Traian Vuia Street, 020956 Bucharest, Romania; gabriel.saramet@umfcd.ro (G.S.)

[7] Department of Pharmaceutical Botany and Cell Biology, Faculty of Pharmacy, "Carol Davila" University of Medicine and Pharmacy, 6 Traian Vuia Street, 020956 Bucharest, Romania; octavian.olaru@umfcd.ro (O.T.O.)

* Correspondence: stefania.barbuceanu@umfcd.ro (S.-F.B.); theodora.apostol@umfcd.ro (T.-V.A.)

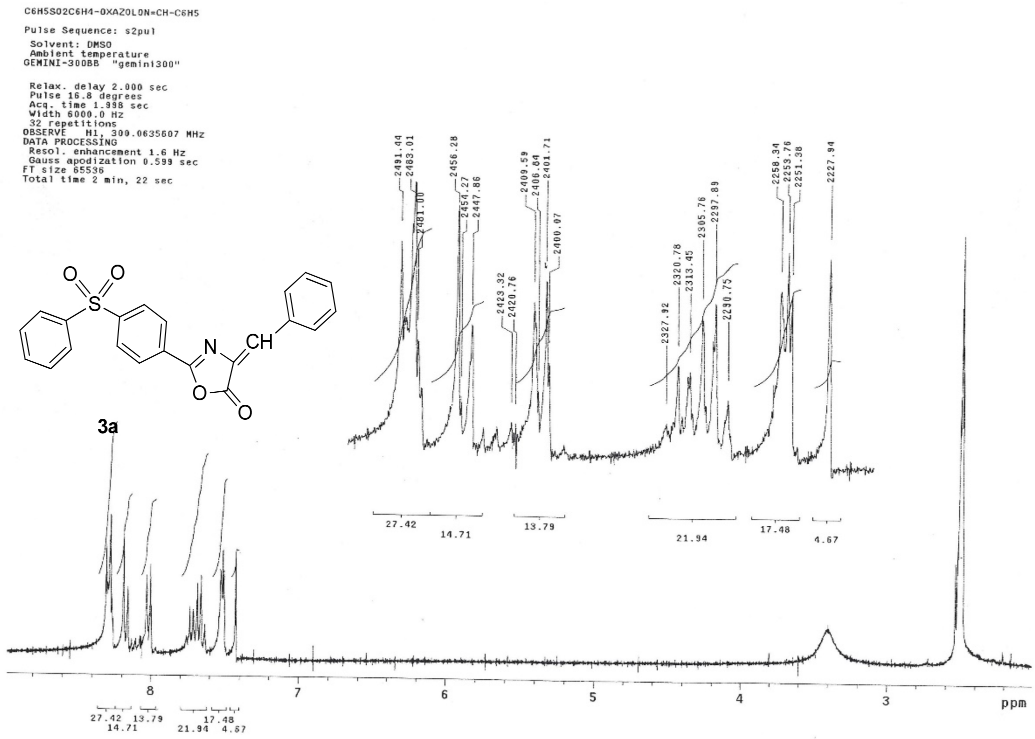

Figure S1. The ^{1}H-NMR spectrum of oxazolone **3a**.

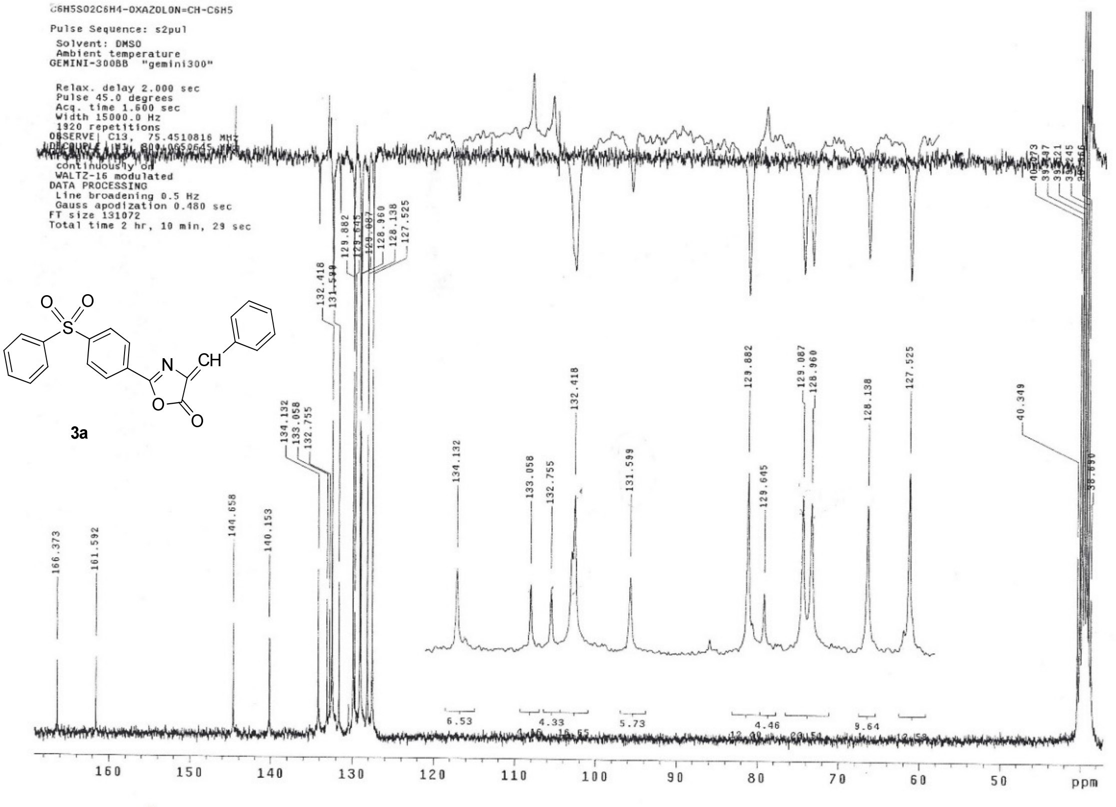

Figure S2. The ^{13}C-NMR spectrum of oxazolone **3a**.

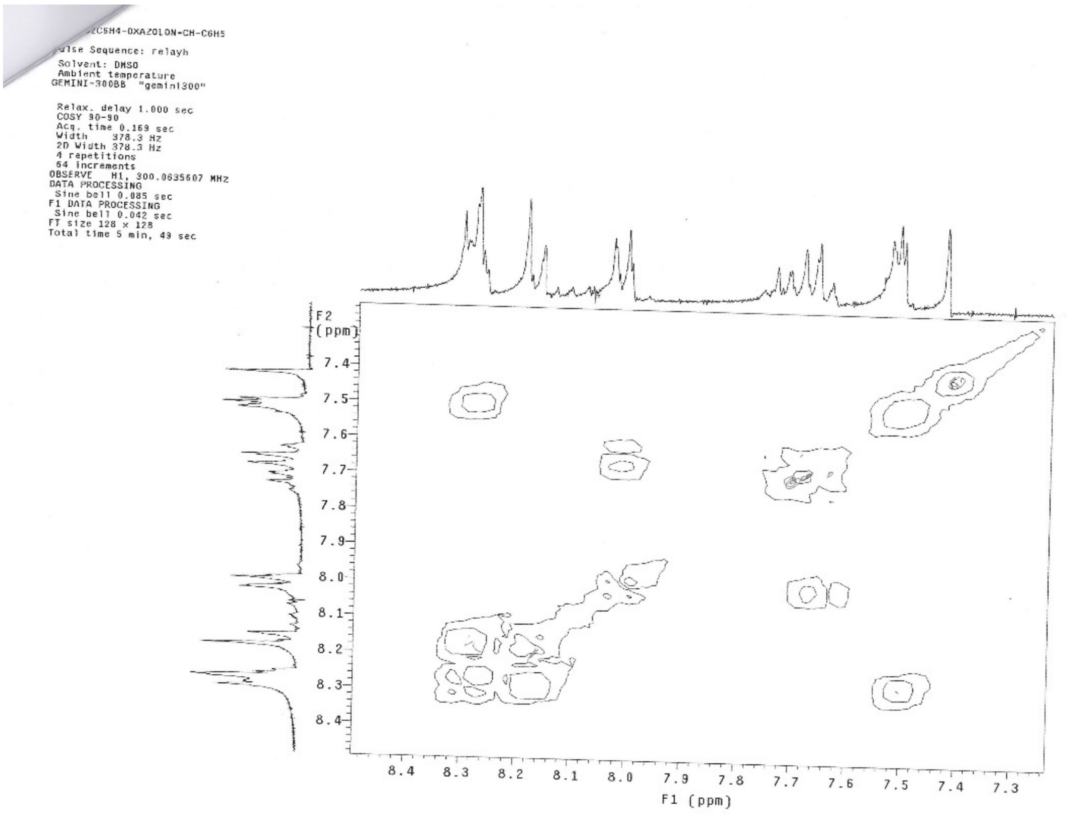

Figure S3. The ^{1}H-^{1}H COSY spectrum of oxazolone **3a**.

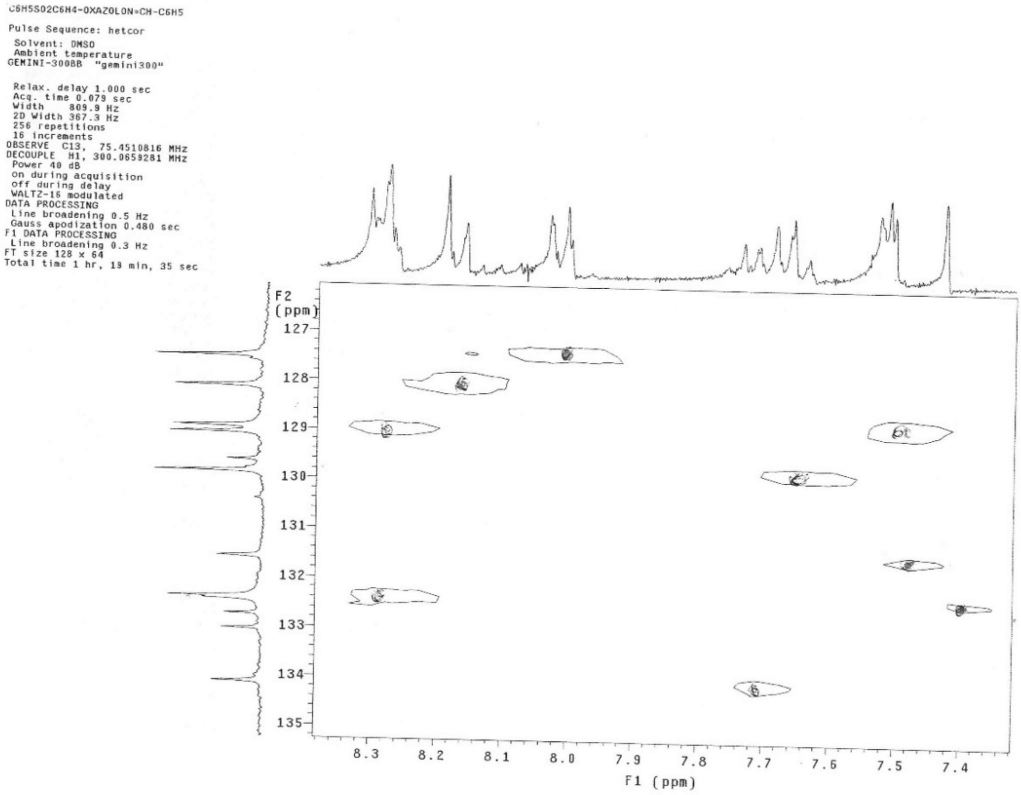

Figure S4. The ^{1}H-^{13}C COSY spectrum of oxazolone **3a**.

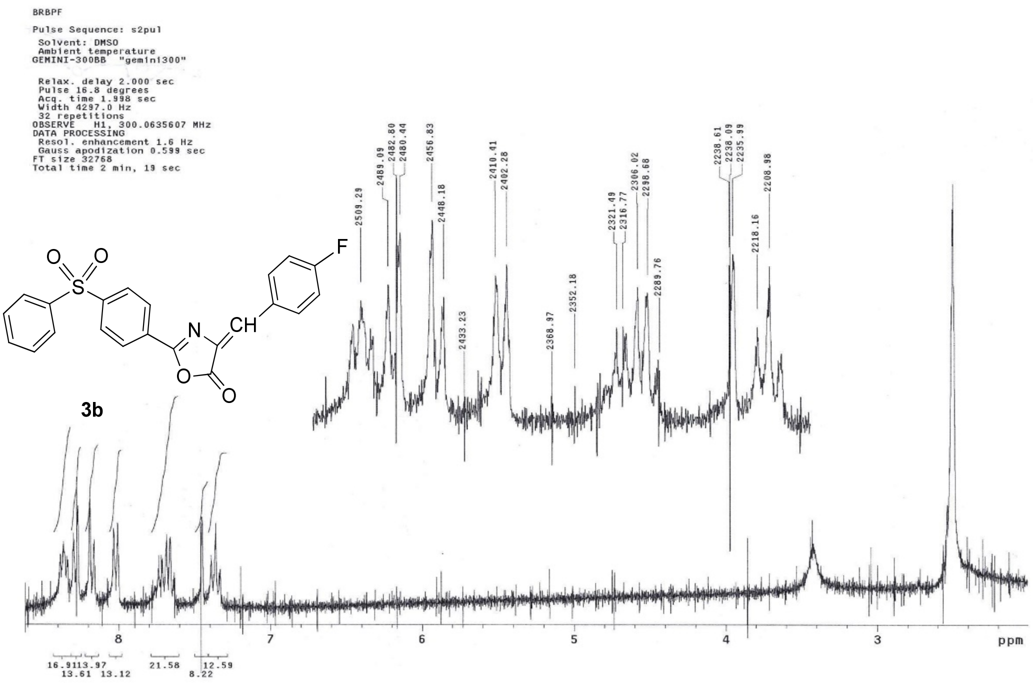

Figure S5. The ¹H-NMR spectrum of oxazolone **3b**.

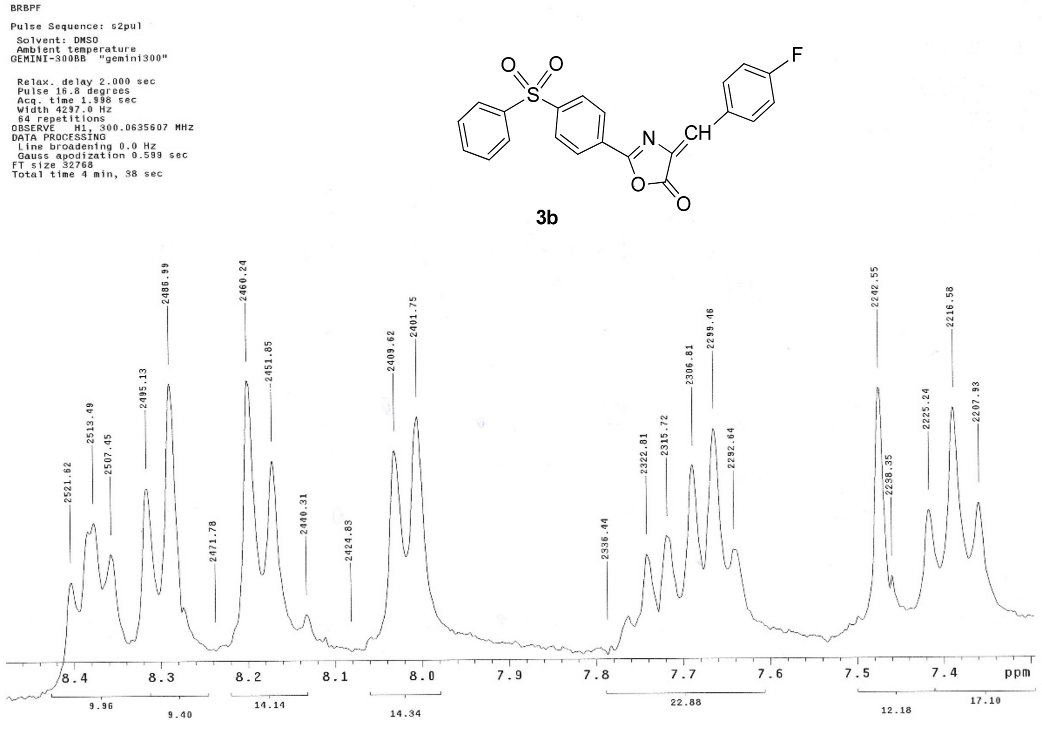

Figure S6. The ¹H-NMR spectrum (detailed) of oxazolone **3b**.

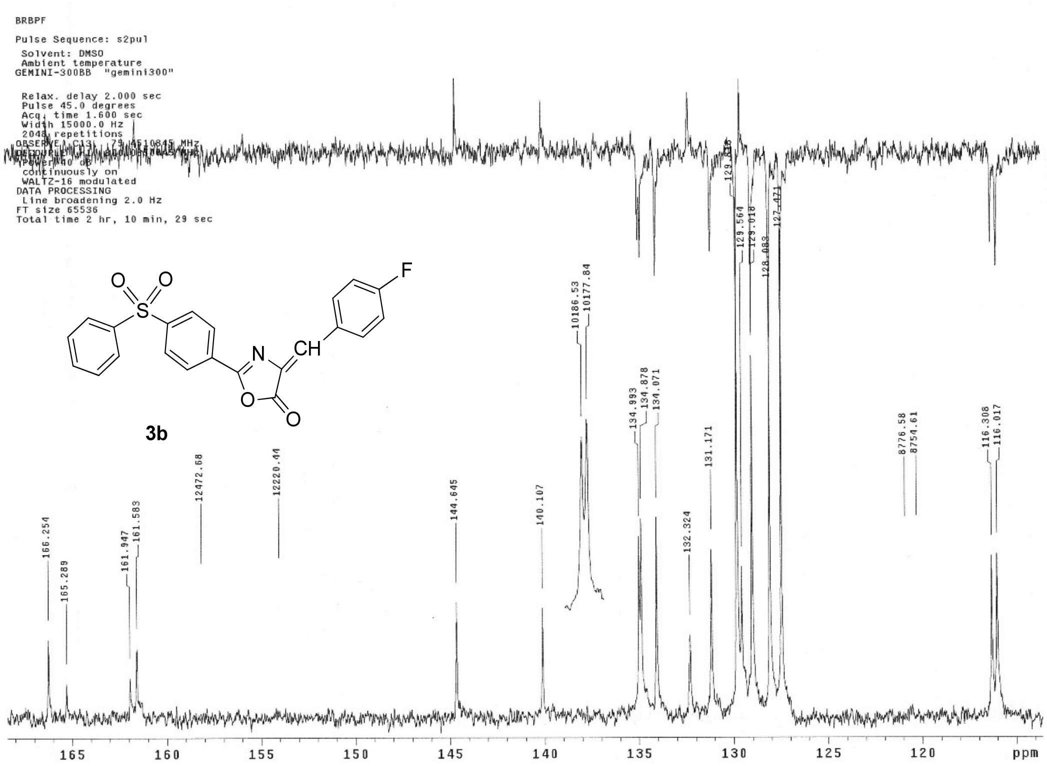

Figure S7. The ^{13}C-NMR spectrum of oxazolone **3b**.

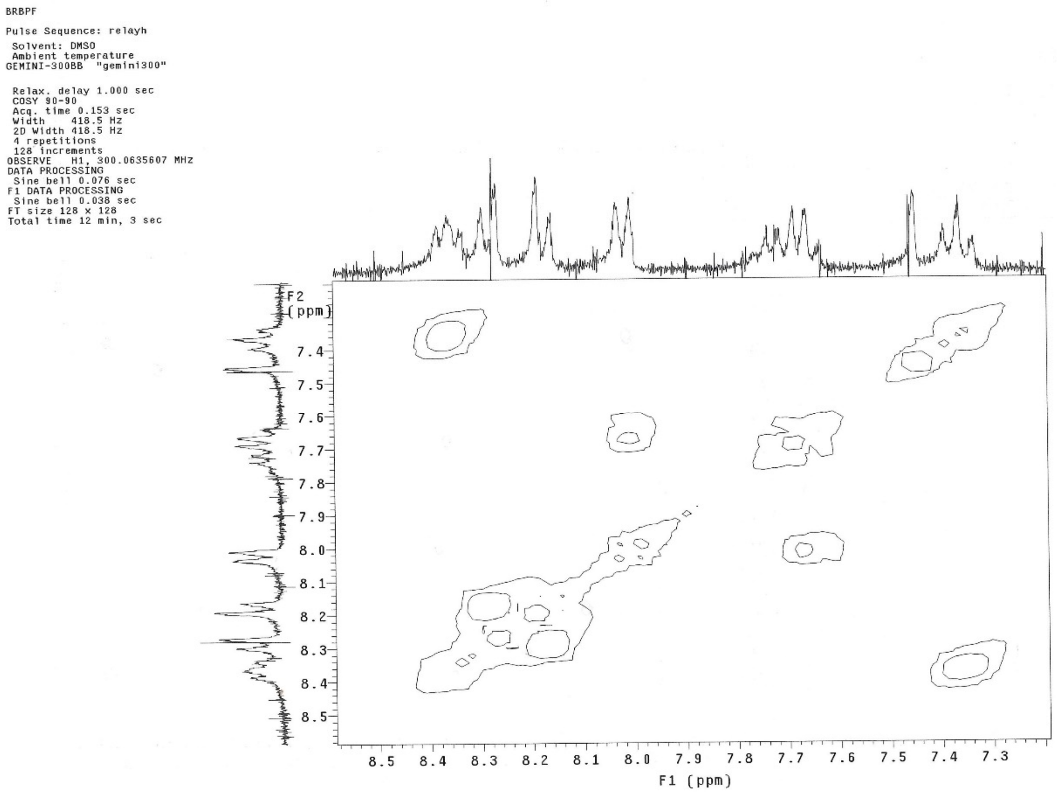

Figure S8. The ^{1}H-^{1}H COSY spectrum of oxazolone **3b**.

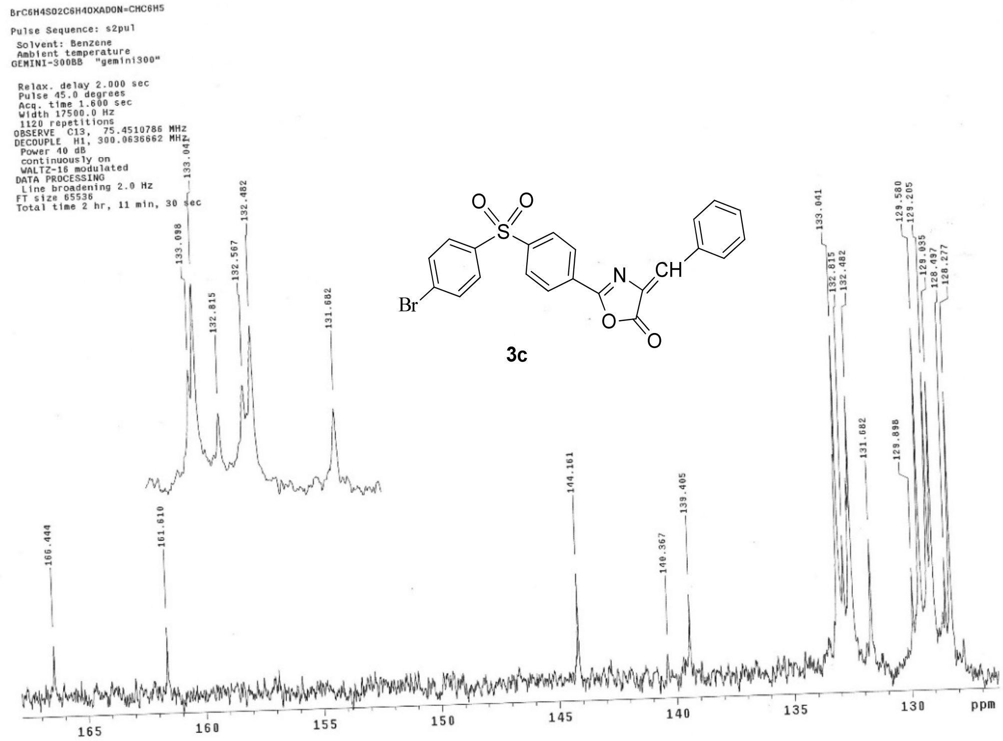

Figure S9. The ^{13}C-NMR spectrum of oxazolone **3c**.

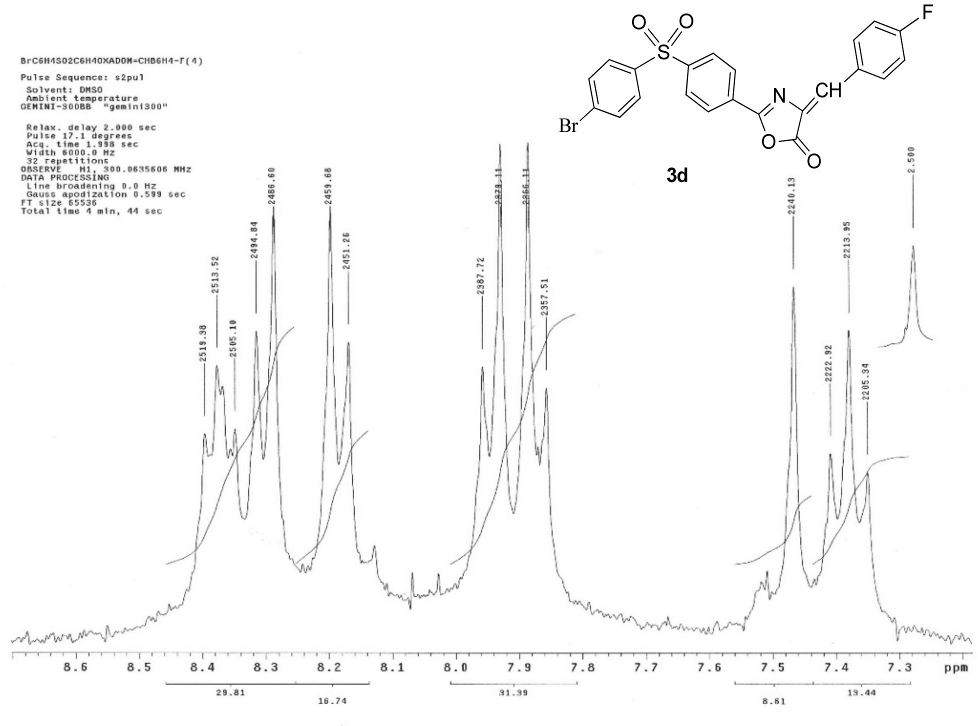

Figure S10. The ^{1}H-NMR spectrum of oxazolone **3d**.

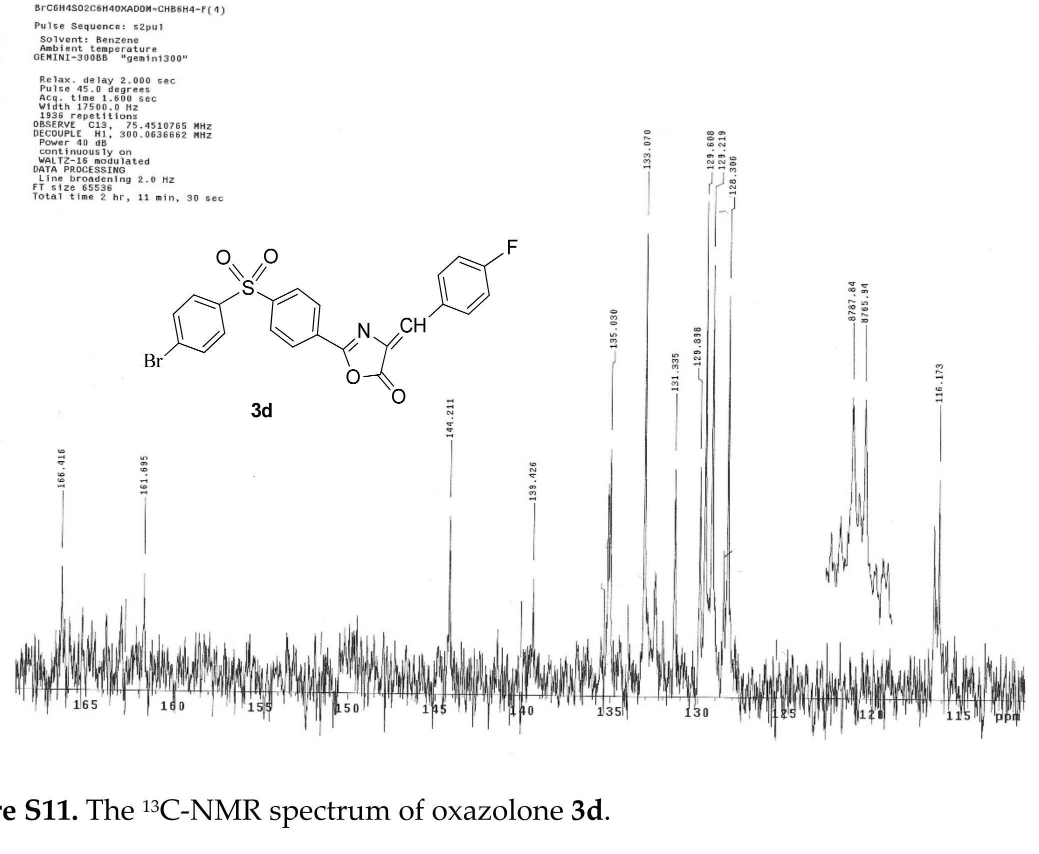

Figure S11. The ^{13}C-NMR spectrum of oxazolone **3d**.

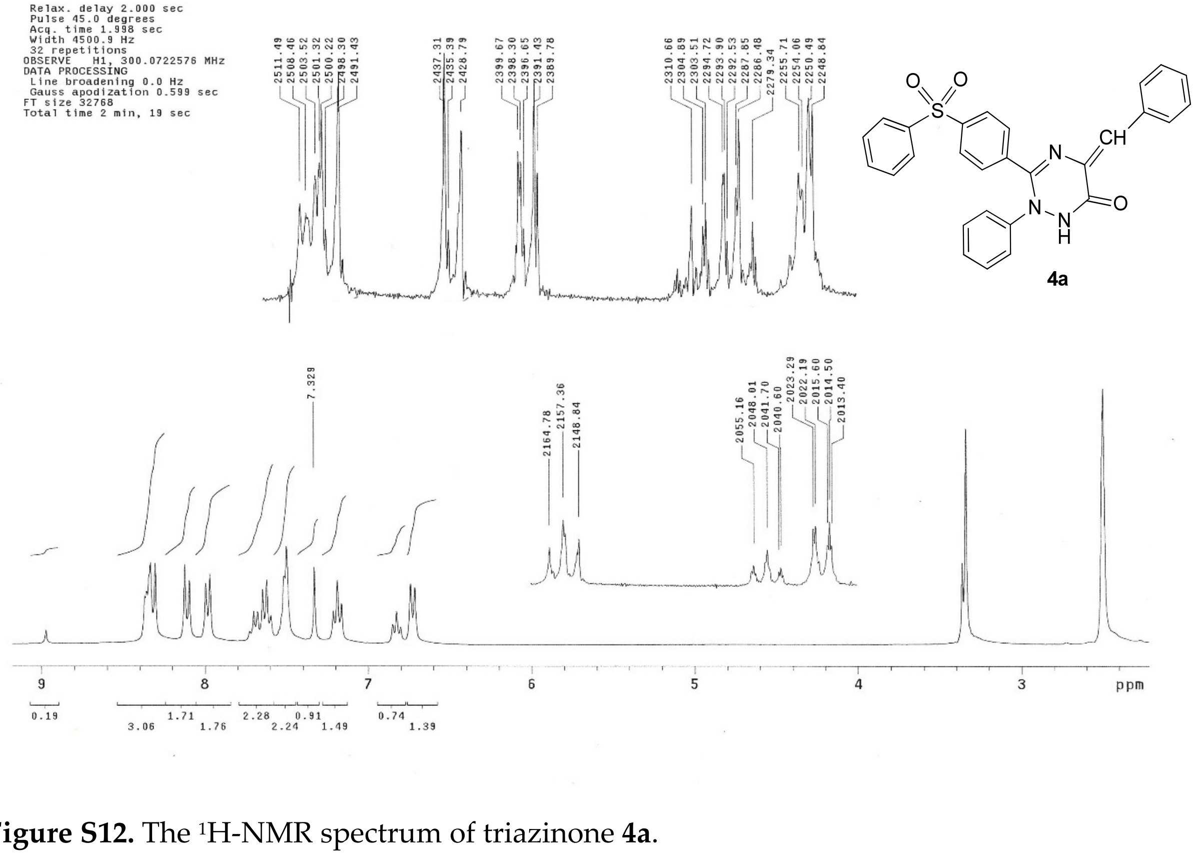

Figure S12. The ^{1}H-NMR spectrum of triazinone **4a**.

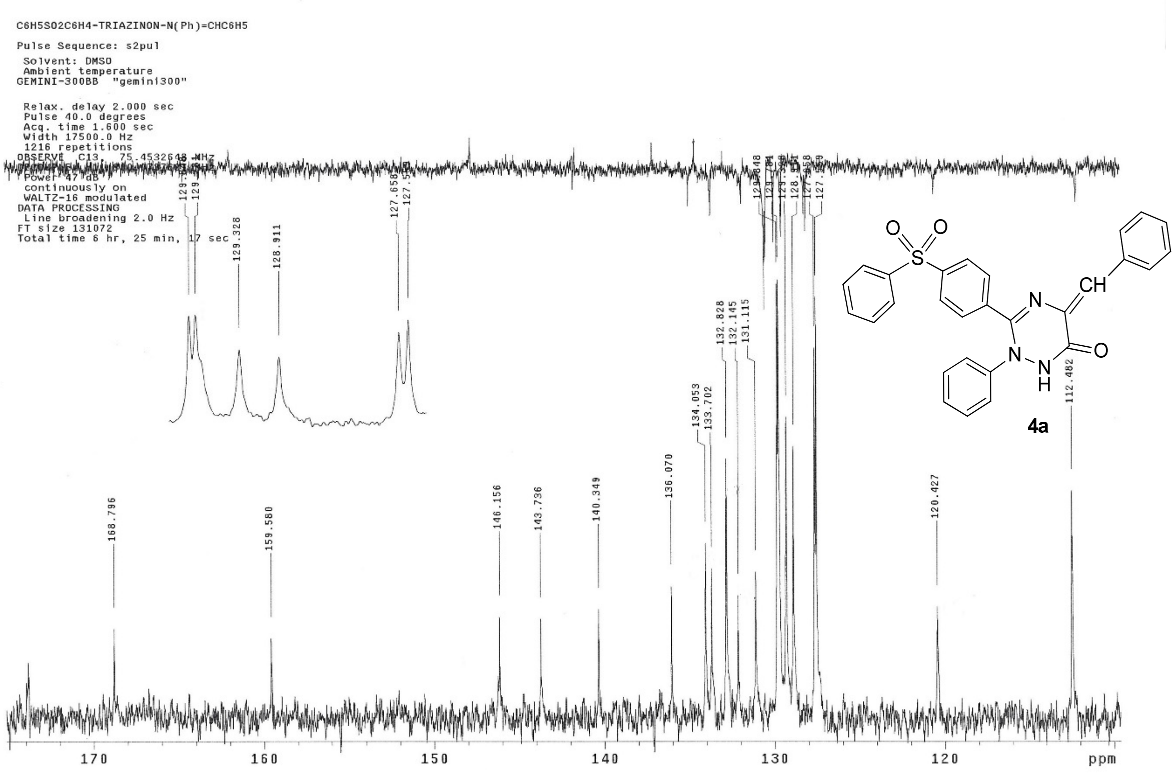

Figure S13. The ^{13}C-NMR spectrum of triazinone **4a**.

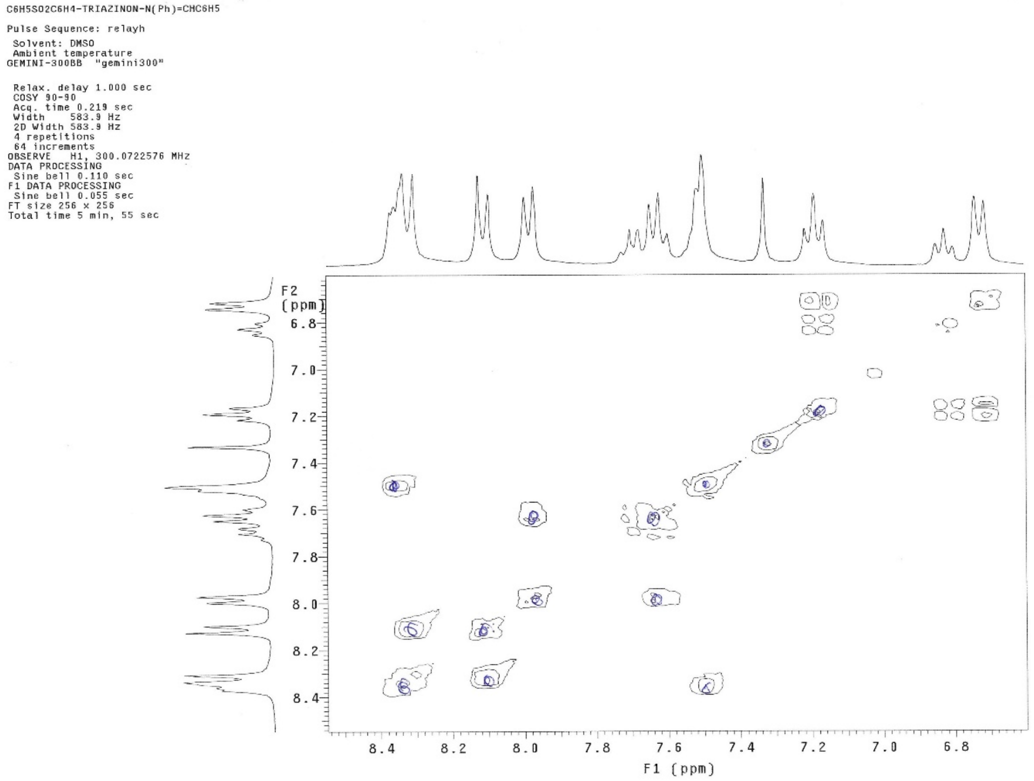

Figure S14. The ^{1}H-^{1}H COSY spectrum of triazinone **4a**.

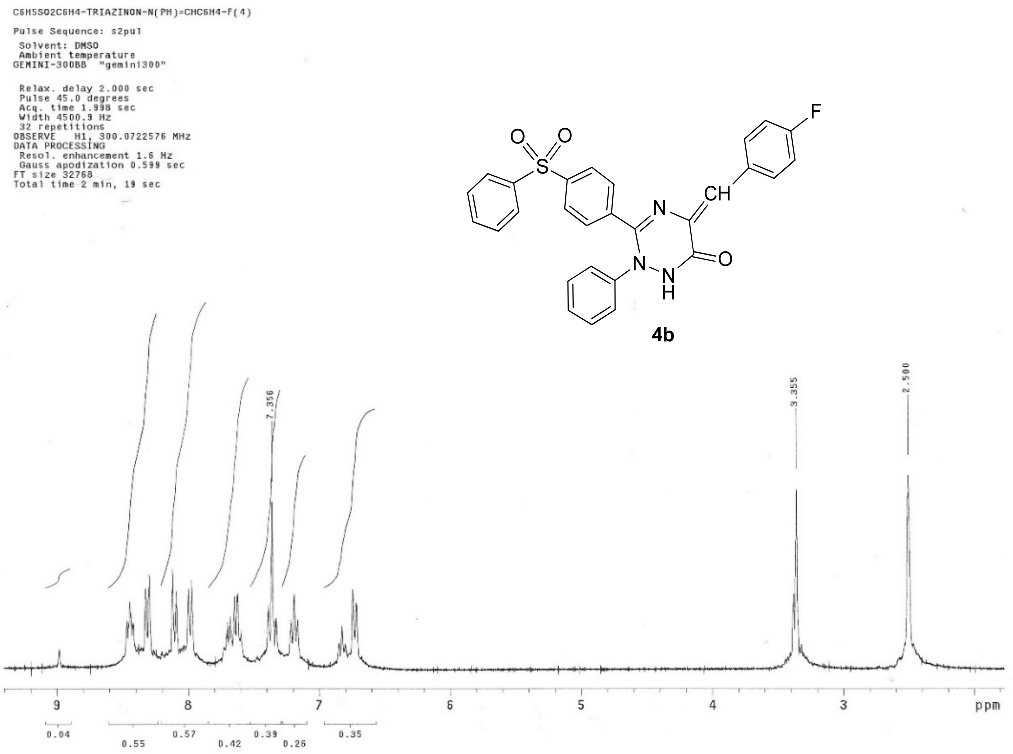

Figure S15. The ¹H-NMR spectrum of triazinone **4b**.

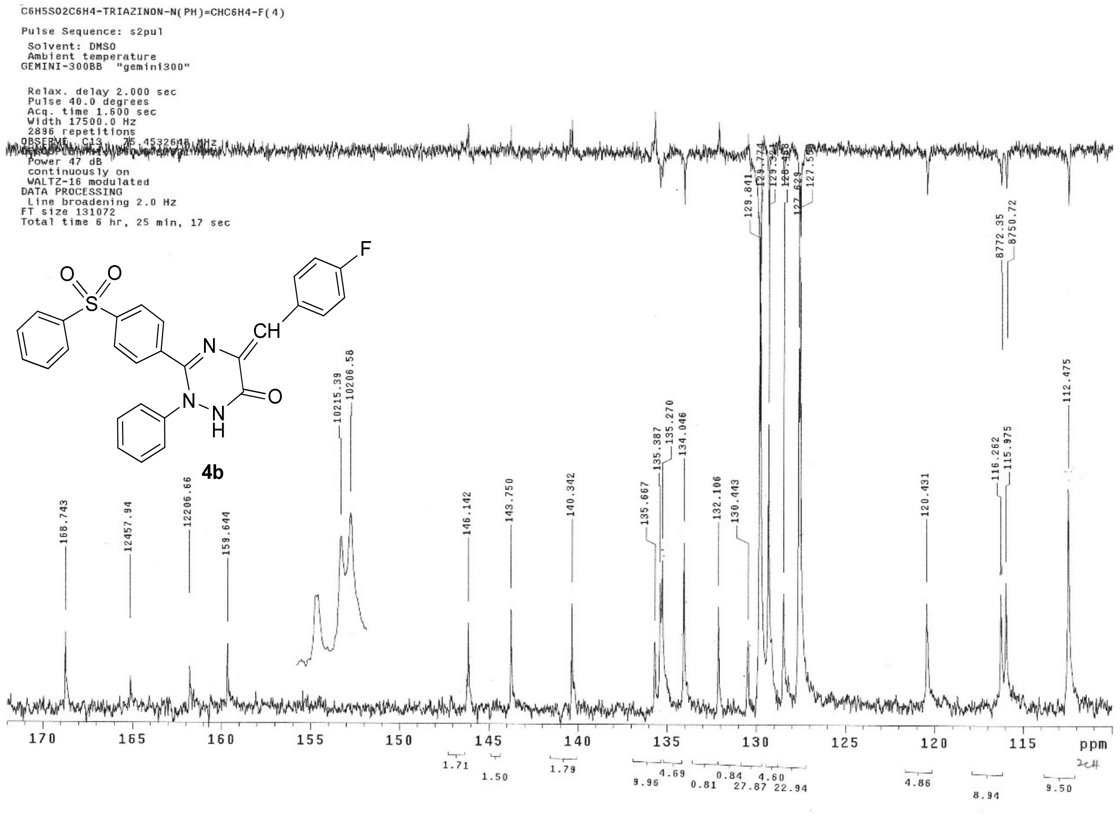

Figure S16. The ¹³C-NMR spectrum of triazinone **4b**.

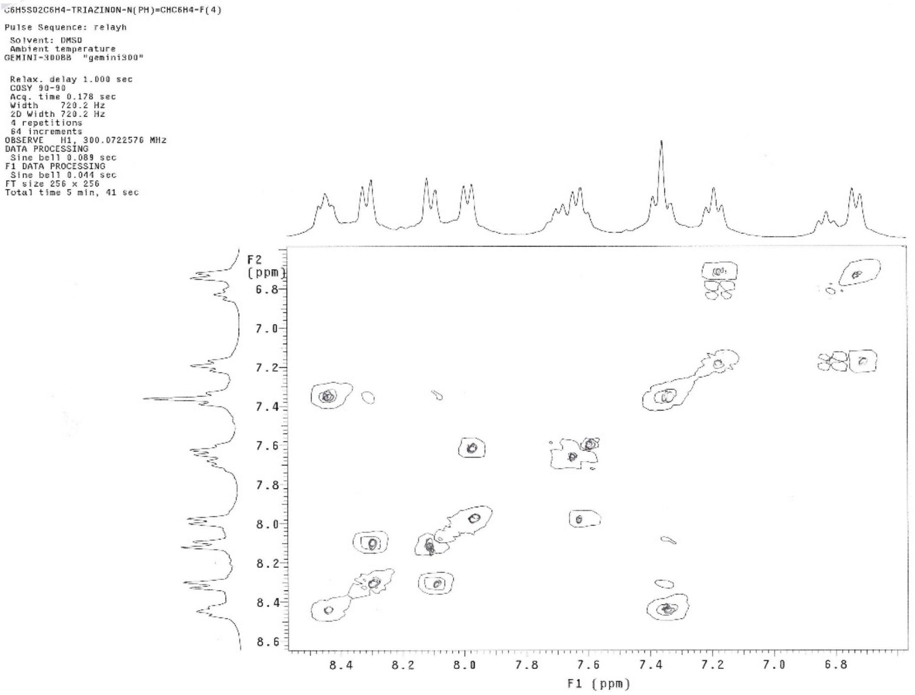

Figure S17. The ^{1}H-^{1}H COSY spectrum of triazinone **4b**.

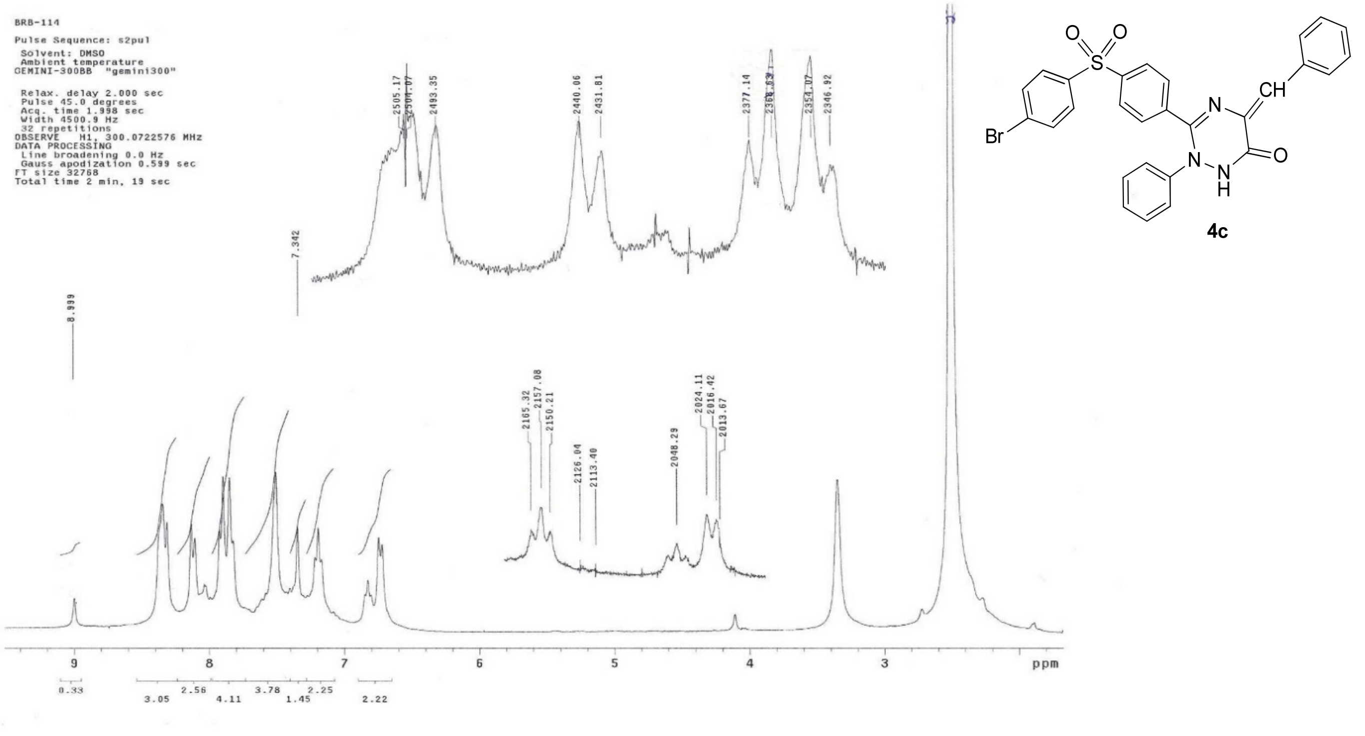

Figure S18. The ^{1}H-NMR spectrum of triazinone **4c**.

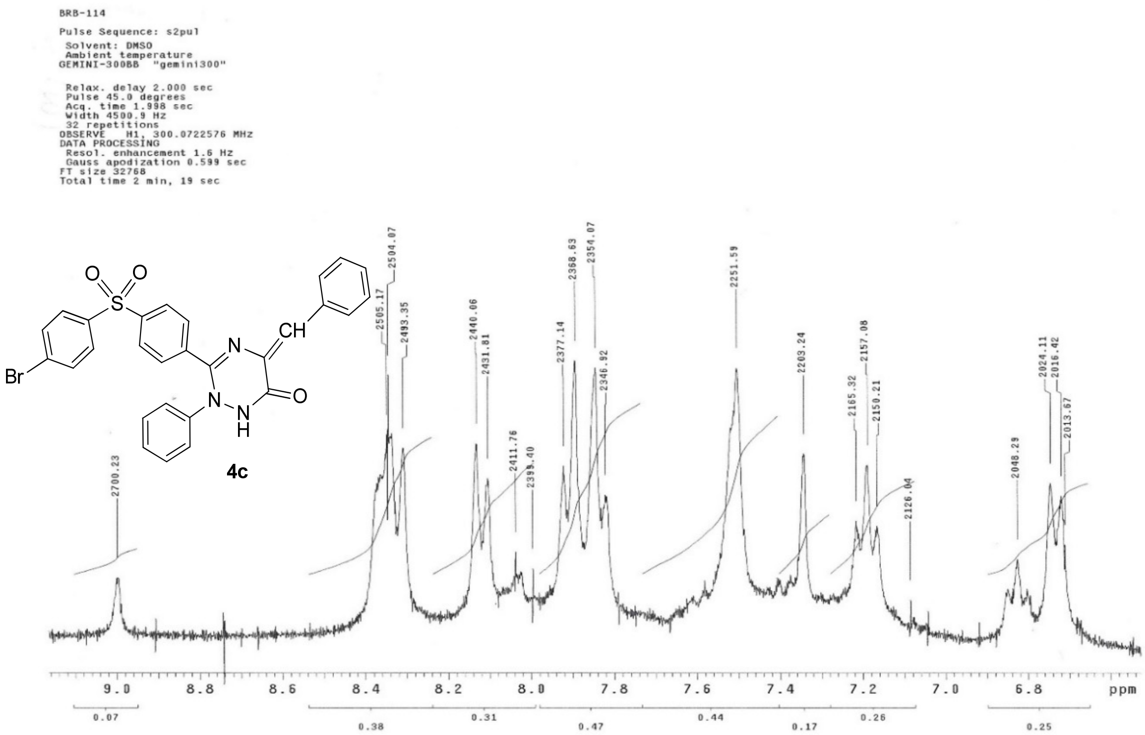

Figure S19. The ^{1}H-NMR spectrum (detailed) of triazinone **4c**.

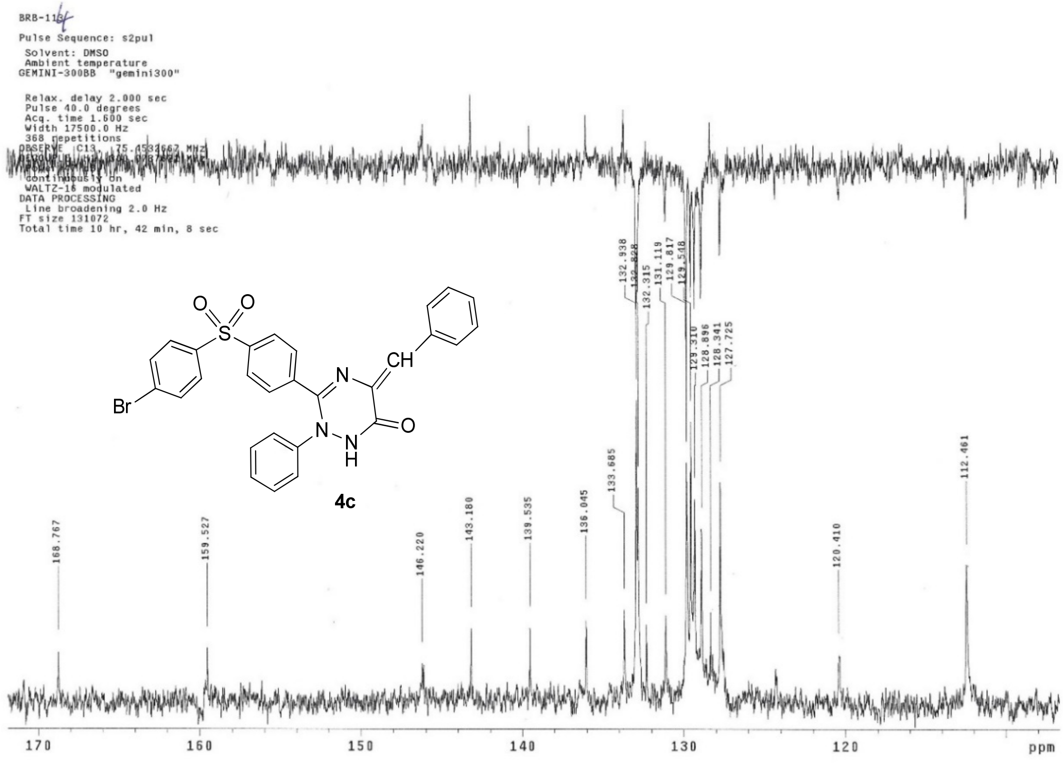

Figure S20. The ^{13}C-NMR spectrum of triazinone **4c**.

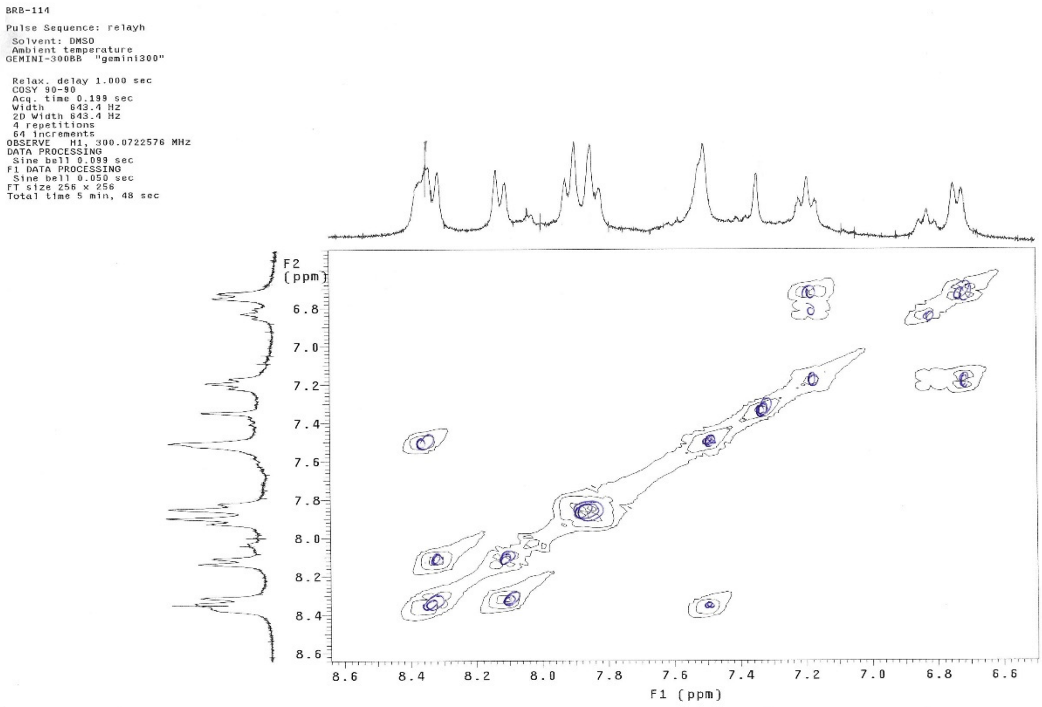

Figure S21. The ^{1}H-^{1}H COSY spectrum of triazinone **4c**.

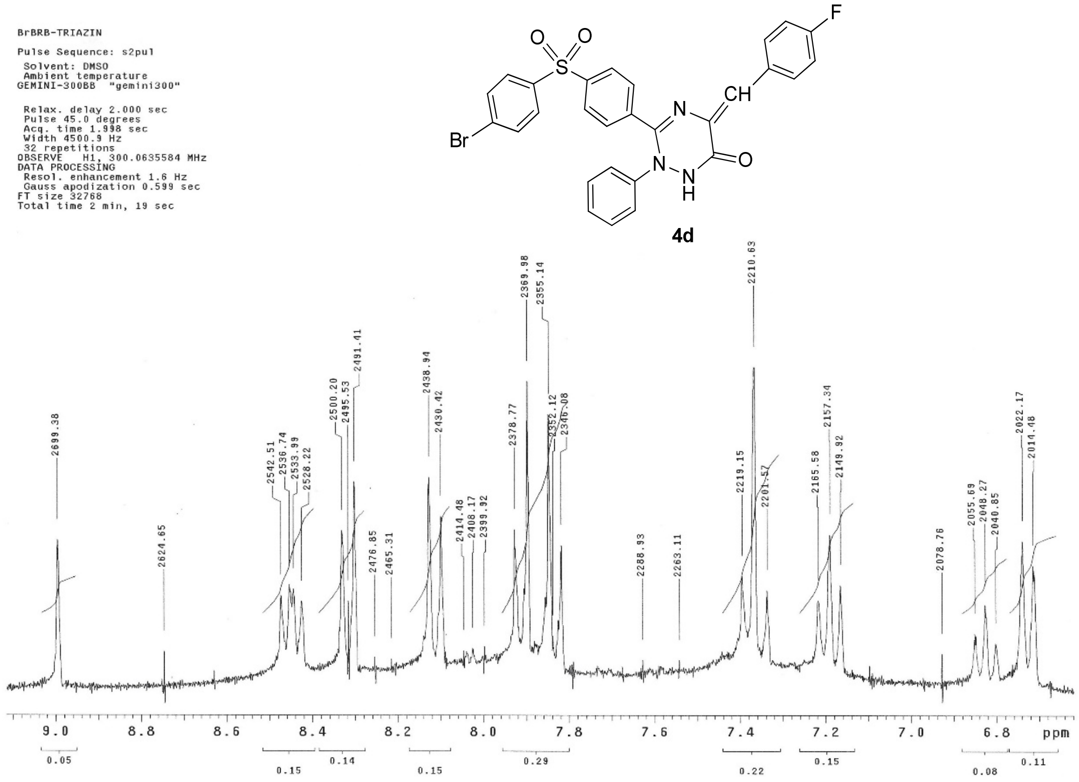

Figure S22. The ^{1}H-NMR spectrum of triazinone **4d**.

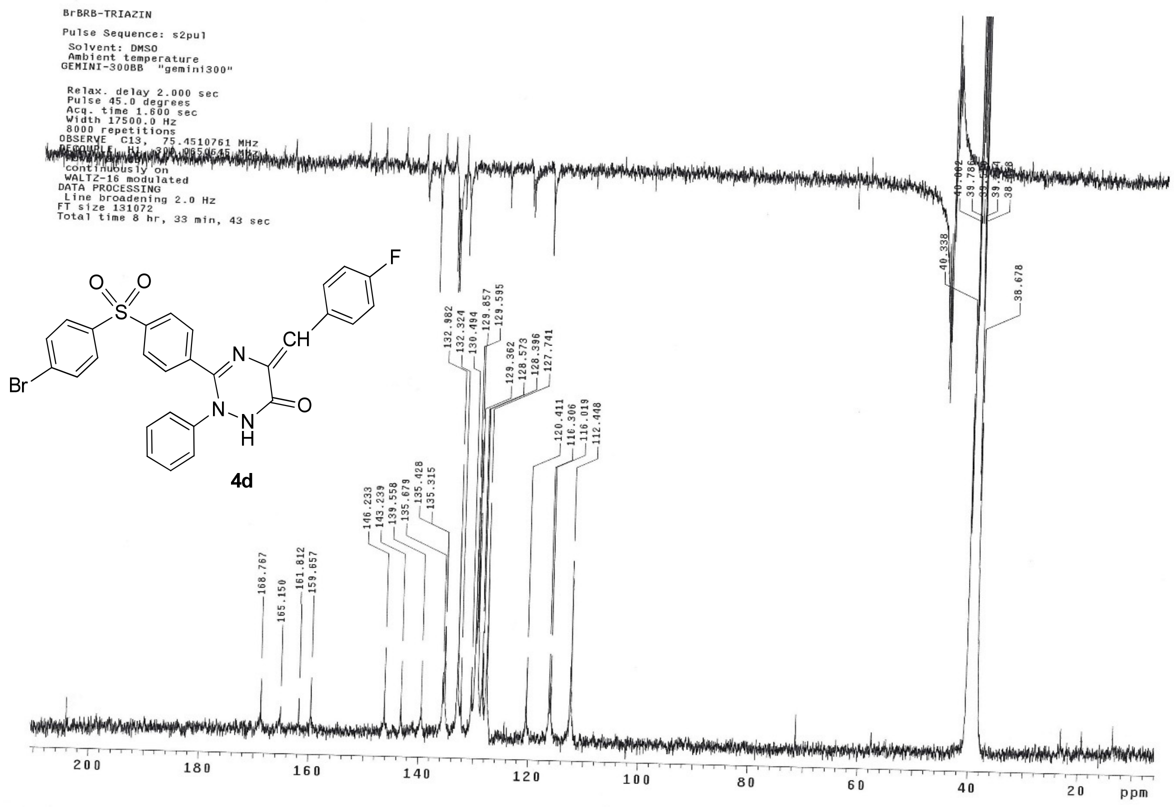

Figure S23. The ^{13}C-NMR spectrum of triazinone **4d**.

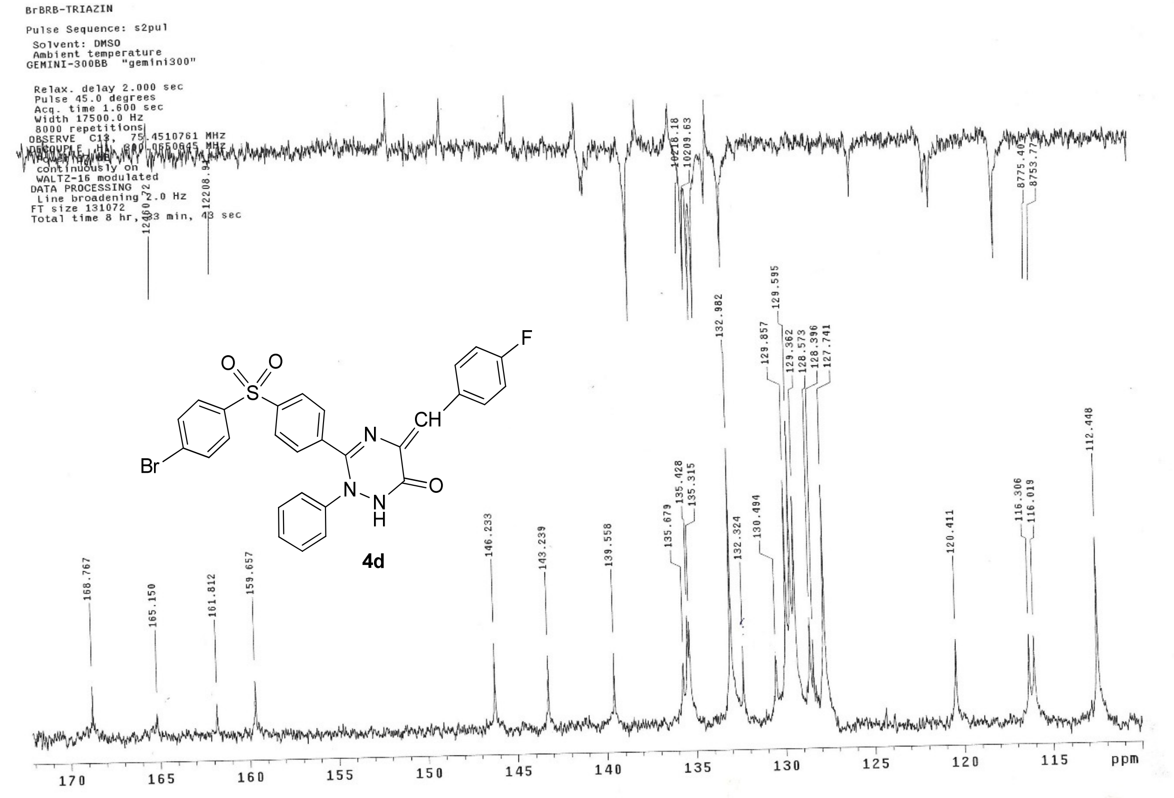

Figure S24. The ^{13}C-NMR spectrum (detailed) of triazinone **4d**.

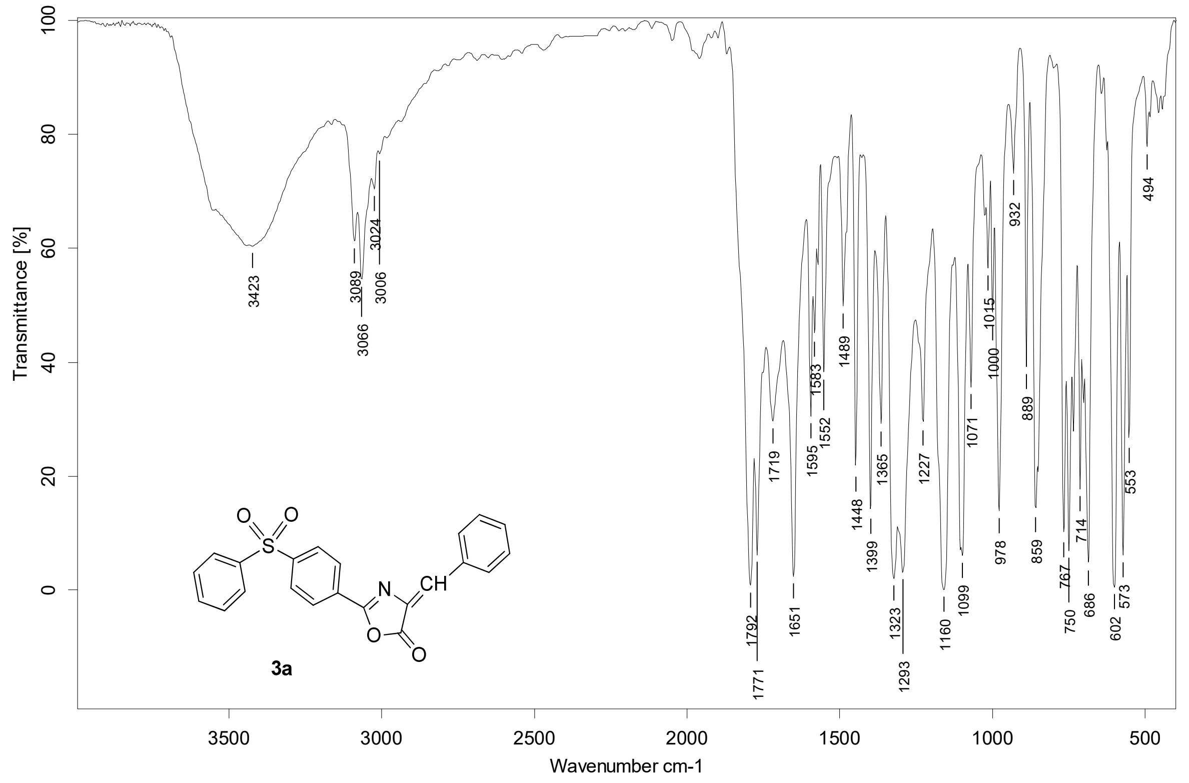

Figure S25. The IR spectrum of oxazol-5(4*H*)-one **3a**.

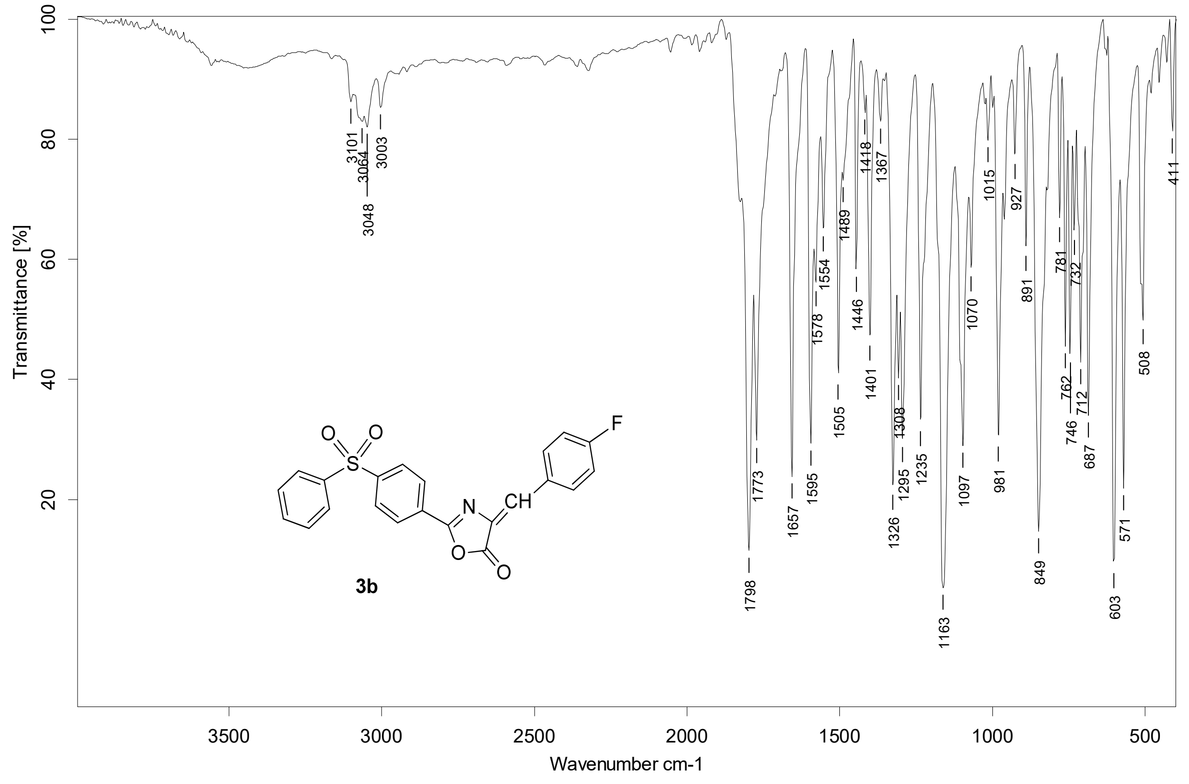

Figure S26. The IR spectrum of oxazol-5(4*H*)-one **3b**.

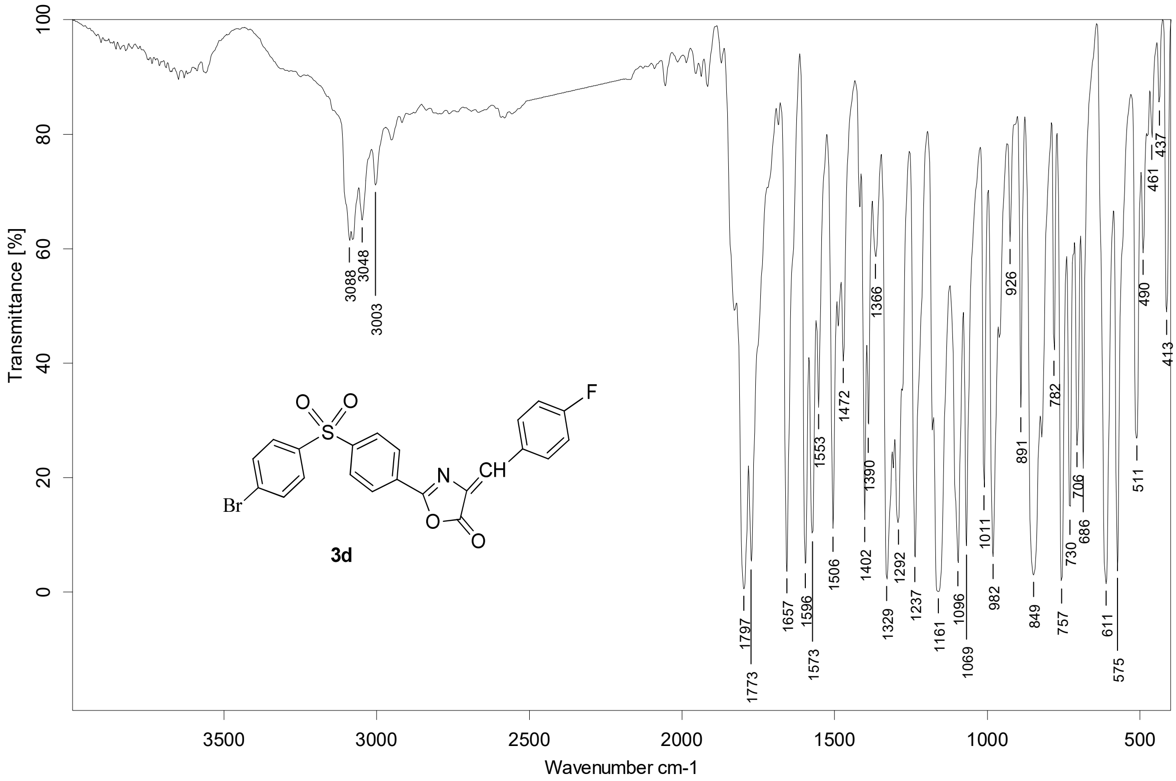

Figure S27. The IR spectrum of oxazol-5(4*H*)-one **3d**.

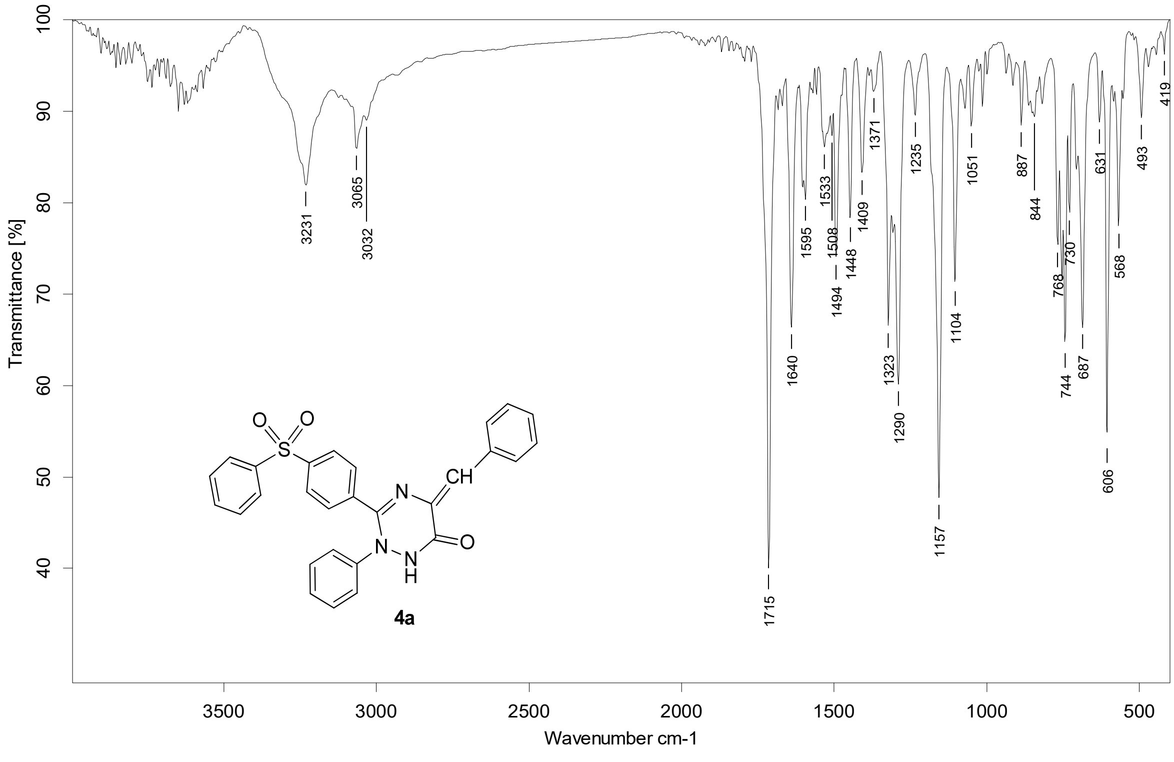

Figure S28. The IR spectrum of triazinone **4a**.

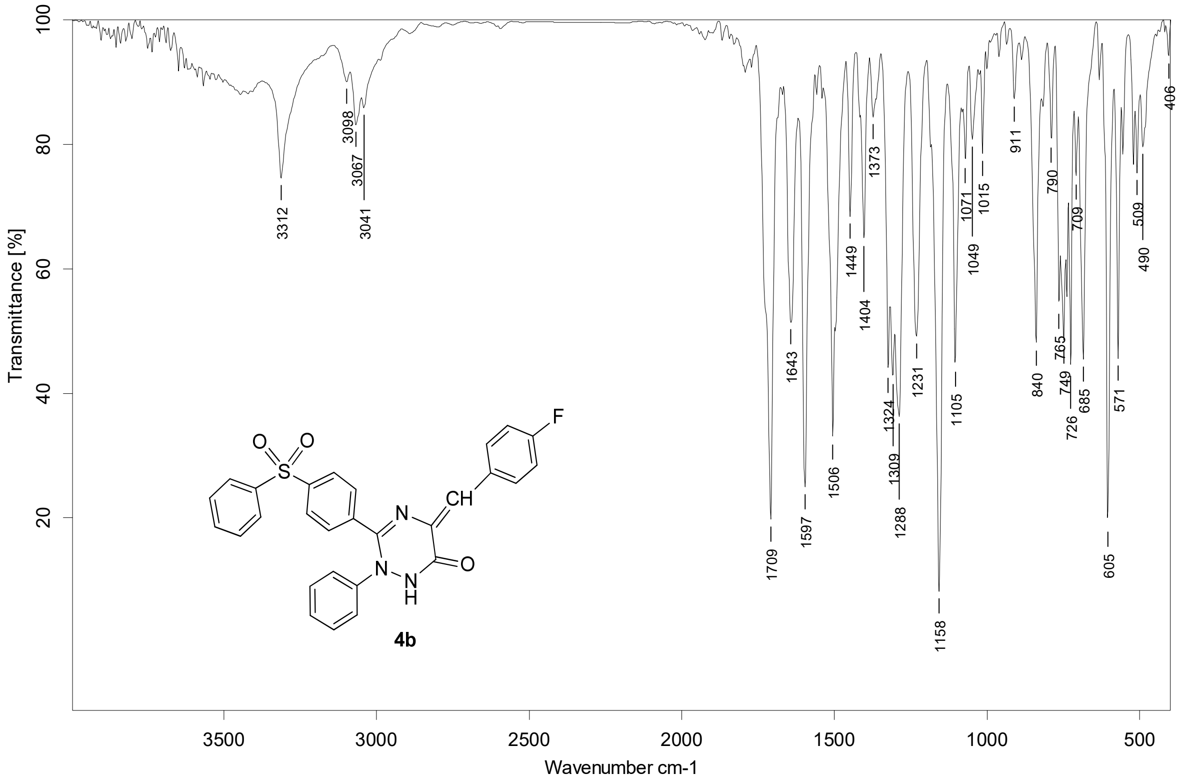

Figure S29. The IR spectrum of triazinone **4b**.

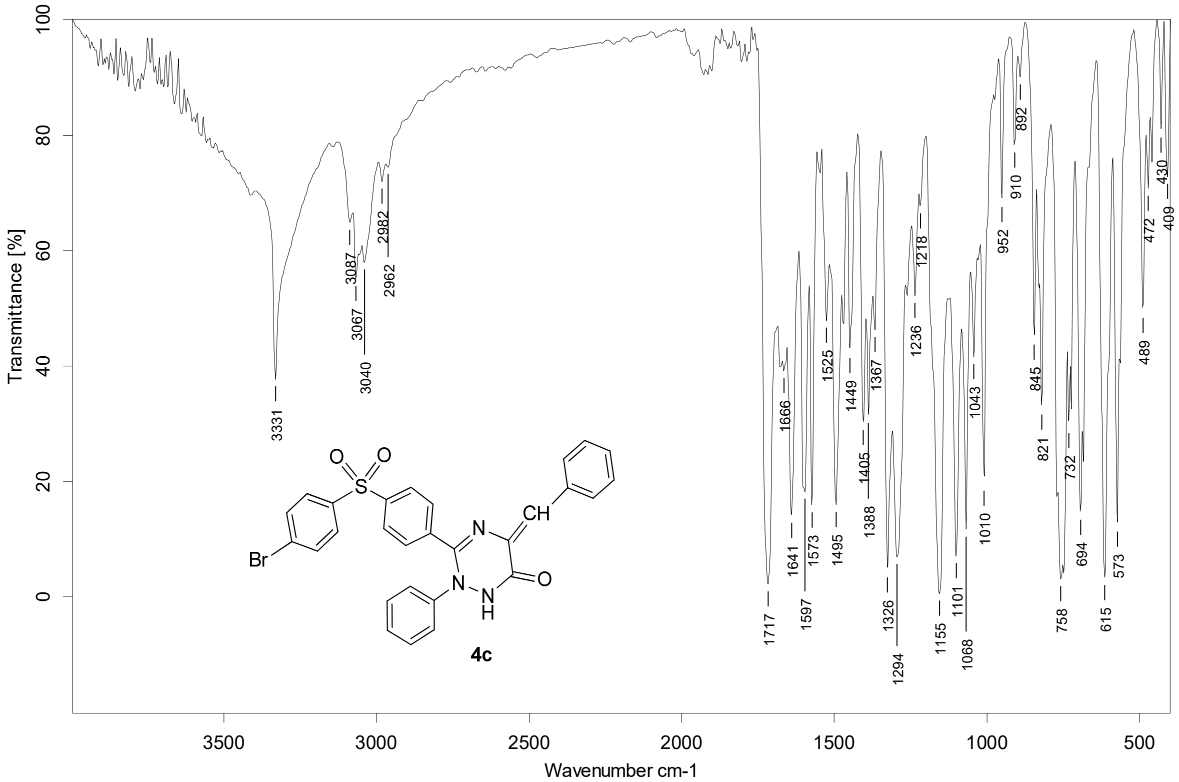

Figure S30. The IR spectrum of triazinone **4c**.

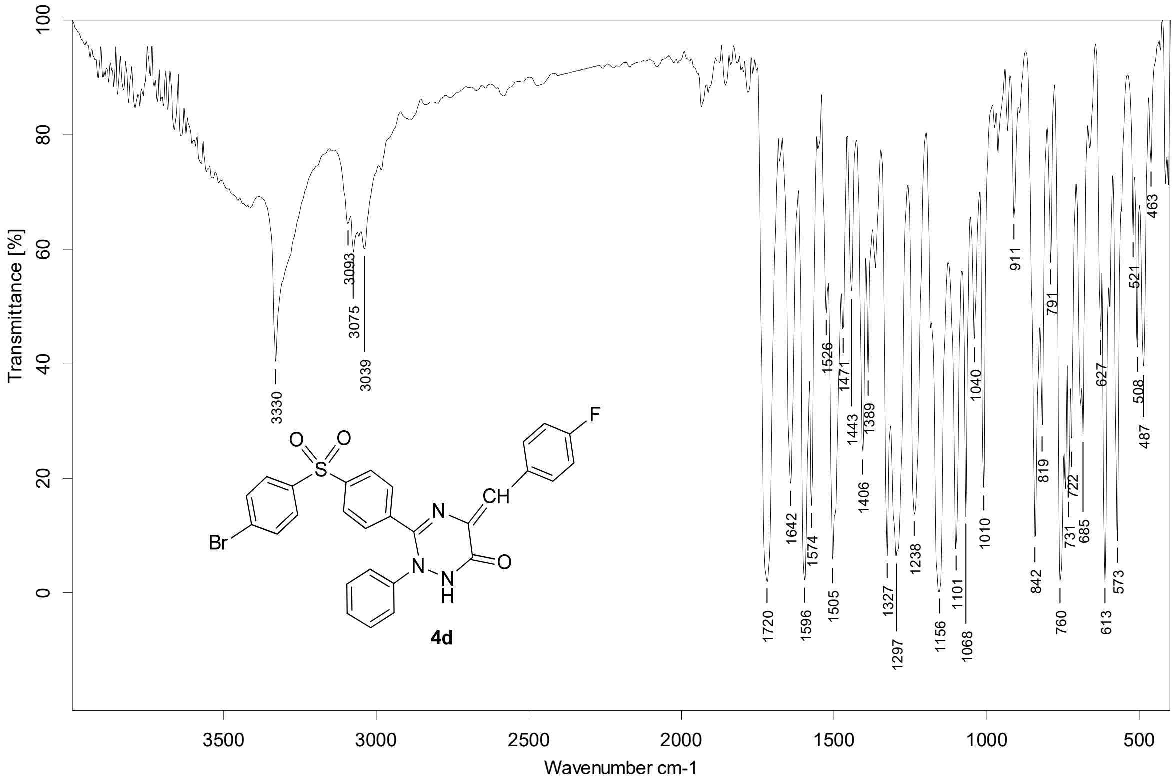

Figure S31. The IR spectrum of triazinone **4d**.

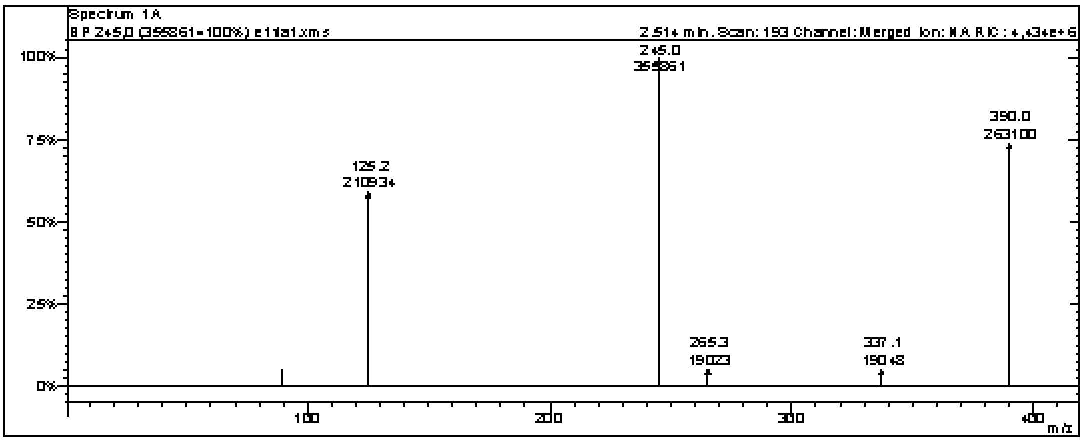

Spectrum from ...\siliceroetari\stefania\e\16-10-2018\e1fla1.xms
Scan No: 193, Time: 2.514 minutes
No averaging. Not background corrected.
Comment: 2.514 min. Scan: 193 Channel: Merged Ion: NA RIC: 4.434e+6
Pair Count: 6 MW: 0 Formula: None
CAS No: None Acquired Range: 20.0 - 400.0 m/z

Method Description: ESI
Scan 1 Channel Description: 390.0 > 20.0 - 400.0 (-10.0 eV)
Scan Information: cp = 0.9 PSI
Precursor Mass Range: 390.0 - 390.0 m/z
Product Mass Range: 20.0 - 400.0 m/z

Ion	Int	%BP	Ion	Int	%BP	Ion	Int	%BP
89.6	18338	5.2	245.0	355861	100.0	337.1	19048	5.4
125.2	210934	59.3	265.3	19023	5.3	390.0	263100	73.9

Figure S32. The +ESI-MS spectra of oxazol-5(4*H*)-one **3a**.

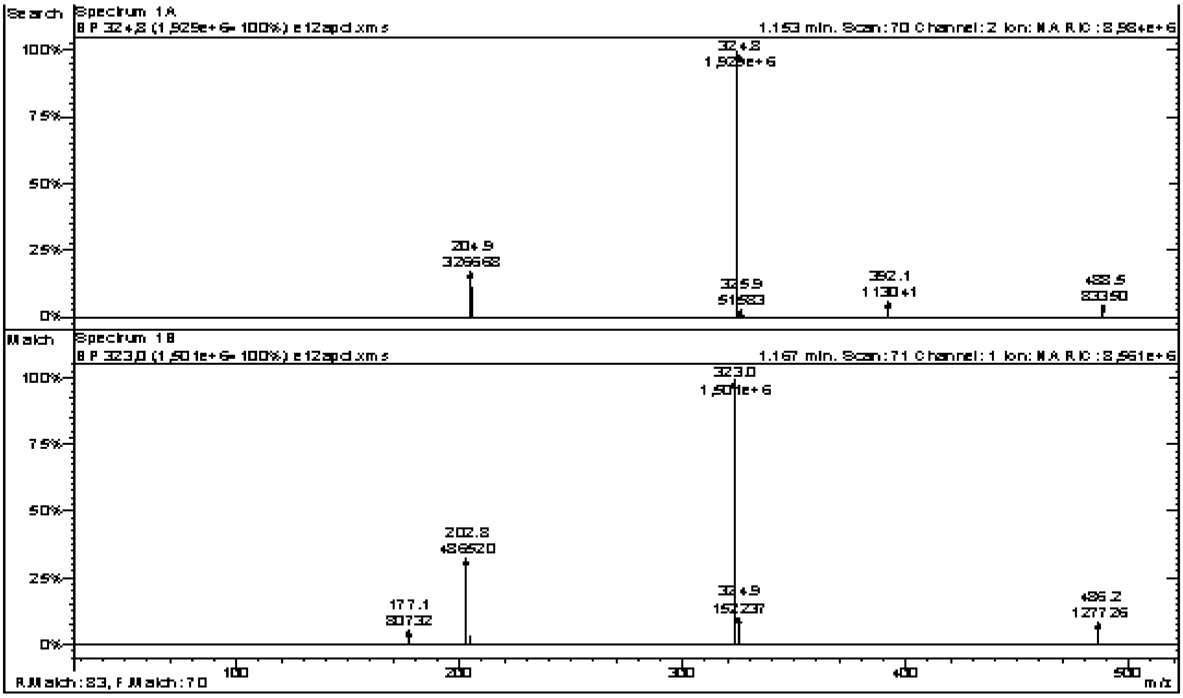

1st Spectrum from ...[illegible]\12-11-2018\e12apci.xms
Scan No: 70, Time: 1.153 minutes
No averaging. Not background corrected.
Comment: 1.153 min. Scan: 70 Channel: 2 Ion: NA RIC: 8,984e+6
Pair Count: 6 MW: 0 Formula: None
CAS No: None Acquired Range: 50.0 - 500.0 m/z

Method Description: APCI
Scan 1 Channel Description: 486.0 > 50.0 - 500.0 (-20.0 eV)
Scan 2 Channel Description: 488.0 > 50.0 - 500.0 (-20.0 eV)
Scan Information: cp = 1.6 PSI
Precursor Mass Range: 488.0 - 488.0 m/z
Product Mass Range: 50.0 - 500.0 m/z

Figure S33. The +APCI-MS spectrum of oxazol-5(4*H*)-one **3d**.

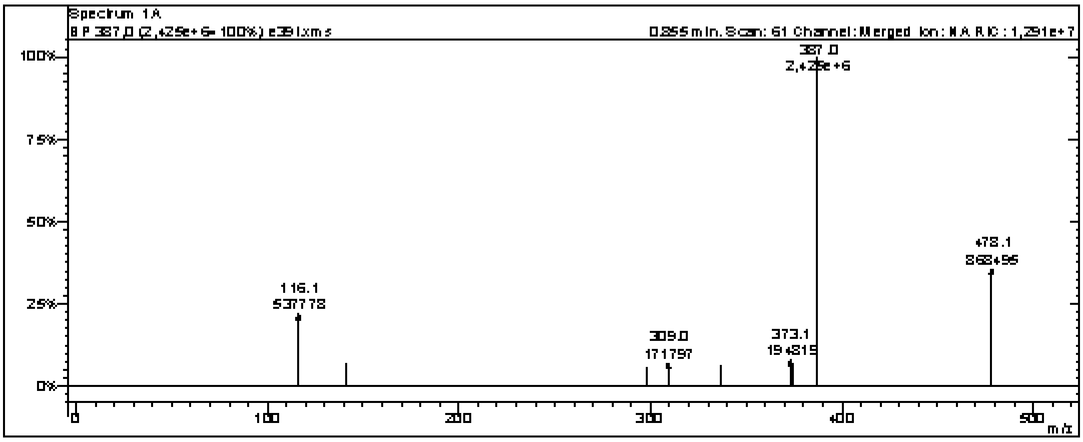

Spectrum from ...d\lisicercetari\stefania\e\11-07-2019\e39t.xms
Scan No: 61, Time: 0.855 minutes
No averaging. Not background corrected.
Comment: 0.855 min. Scan: 61 Channel: Merged Ion: NA RIC: 1,291e+7
Pair Count: 9 MW: 0 Formula: None
CAS No: None Acquired Range: 20.0 - 500.0 m/z

Method Description: APCI
Scan 1 Channel Description: 478.0 > 20.0 - 500.0 (30.0 eV)
Scan Information: cp = 1.4 PSI
Precursor Mass Range: 478.0 - 478.0 m/z
Product Mass Range: 20.0 - 500.0 m/z

Ion	Int	%BP	Ion	Int	%BP	Ion	Int	%BP
116.1	537778	22.2	309.0	171797	7.1	374.2	162592	6.7
140.8	173651	7.2	337.1	161242	6.7	387.0	2,425e+6	100.0
298.0	148674	6.1	373.1	194819	8.0	478.1	868495	35.8

Figure S34. The –APCI-MS spectrum of triazinone **4a**.

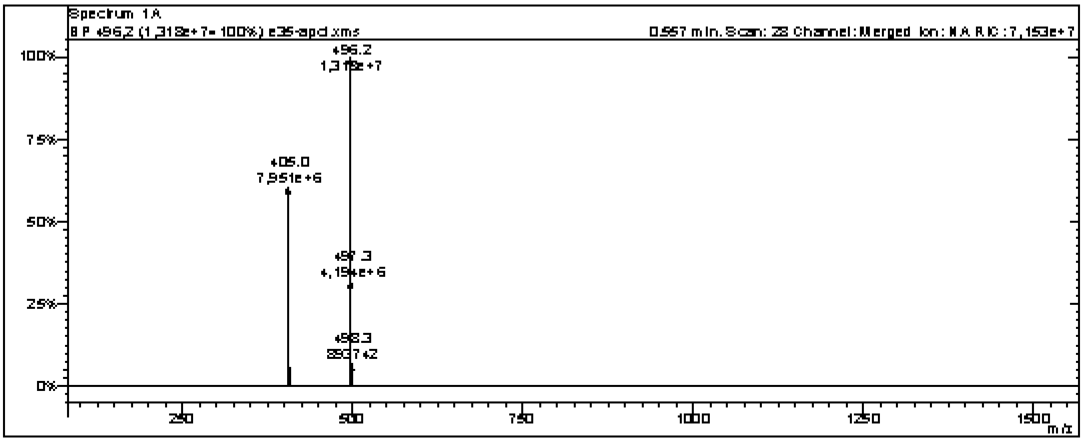

Spectrum from ...s\cercetari\stefania\e\25-06-2019\e35-apci.xms
Scan No: 28, Time: 0.557 minutes
No averaging. Not background corrected.
Comment: 0.557 min. Scan: 28 Channel: Merged Ion: NA RIC: 7,153e+7
Pair Count: 6 MW: 0 Formula: None
CAS No: None Acquired Range: 150.0 - 1500.0 m/z

Method Description: APCI
Scan 1 Channel Description: 150.0 - 1500.0 >
Scan Information: cp = 0.0 PSI
Precursor Mass Range: 150.0 - 1500.0 m/z

Ion	Int	%BP	Ion	Int	%BP	Ion	Int	%BP
405.0	7,951e+6	60.3	407.2	740672	5.6	497.3	4,194e+6	31.8
406.0	2,649e+6	20.1	496.2	1,318e+7	100.0	498.3	893742	6.8

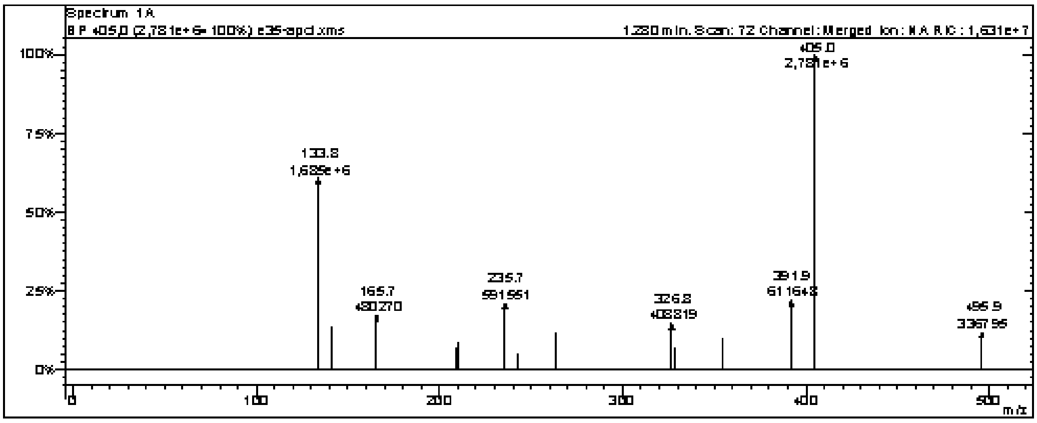

Spectrum from ...s\cercetari\stefania\e\25-06-2019\e35-apci.xms
Scan No: 72, Time: 1.280 minutes
No averaging. Not background corrected.
Comment: 1.280 min. Scan: 72 Channel: Merged Ion: NA RIC: 1,631e+7
Pair Count: 14 MW: 0 Formula: None
CAS No: None Acquired Range: 20.0 - 500.0 m/z

Method Description: APCI
Scan 1 Channel Description: 496.0 > 20.0 - 500.0 (40.0 eV)
Scan Information: cp = 1.5 PSI
Precursor Mass Range: 496.0 - 496.0 m/z
Product Mass Range: 20.0 - 500.0 m/z

Ion	Int	%BP	Ion	Int	%BP	Ion	Int	%BP
133.8	1,685e+6	60.6	235.7	591551	21.3	354.9	278525	10.0
140.9	384469	13.8	242.4	151607	5.5	391.9	611648	22.0
165.7	480270	17.3	263.9	338391	12.2	405.0	2,781e+6	100.0
209.3	197649	7.1	326.8	408819	14.7	495.9	336795	12.1
210.2	252328	9.1	327.9	205470	7.4			

Figure S35. The –APCI-MS spectrum of triazinone **4b**.

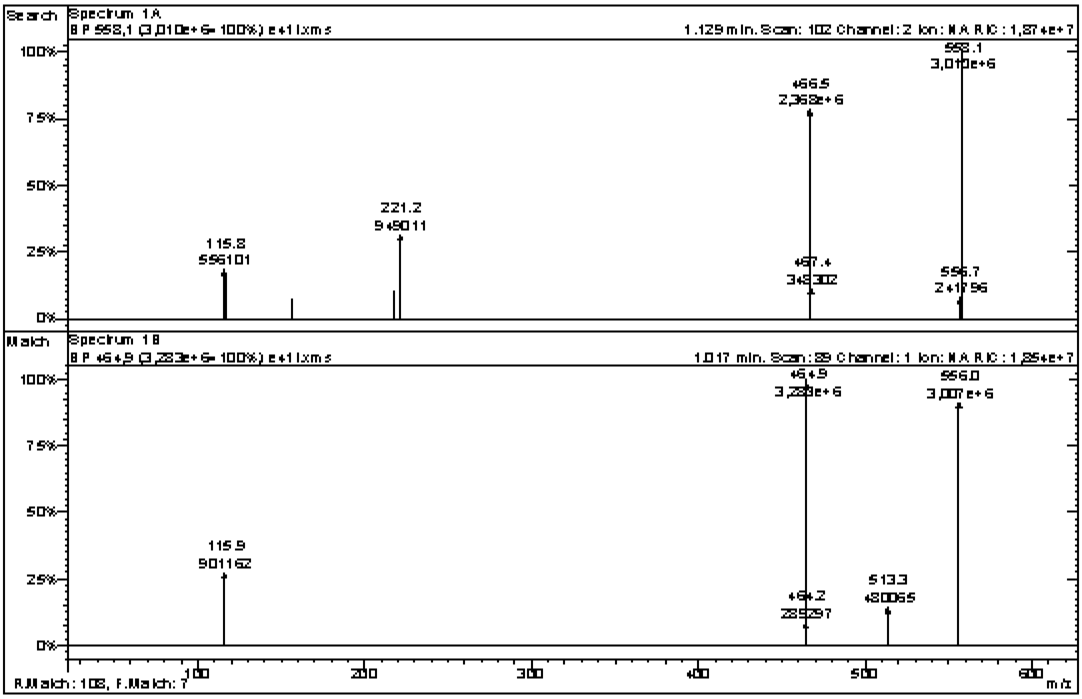

1st Spectrum from ...[illegible]\11-07-2019\[illegible]
Scan No: 102, Time: 1.129 minutes
No averaging. Not background corrected.
Comment: 1.129 min. Scan: 102 Channel: 2 Ion: NA RIC: 1,874e+7
Pair Count: 9 MW: 0 Formula: None
CAS No: None Acquired Range: 50.0 - 600.0 m/z

Method Description: APCI
Scan 1 Channel Description: 556.0 > 50.0 - 600.0 (30.0 eV)
Scan 2 Channel Description: 558.0 > 50.0 - 600.0 (30.0 eV)

Figure S36. The –APCI-MS spectrum of triazinone **4c**.

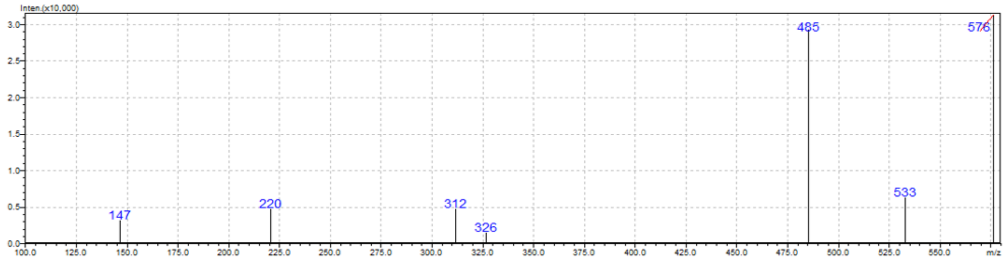

[MS Spectrum]

# of Peaks	7	
Raw Spectrum	[0.705] (scan:[283])	
Background	No Background Spectrum	
Precursor	576.00	
Base Peak	m/z 576.15 (Inten : 31,271)	
m/z	Absolute Intensity	Relative Intensity
146.70	3221	10.30
220.50	4827	15.44
311.60	4827	15.44
326.40	1609	5.15
485.05	29336	93.81
532.70	6452	20.63
576.15	31271	100.00
Event	1	

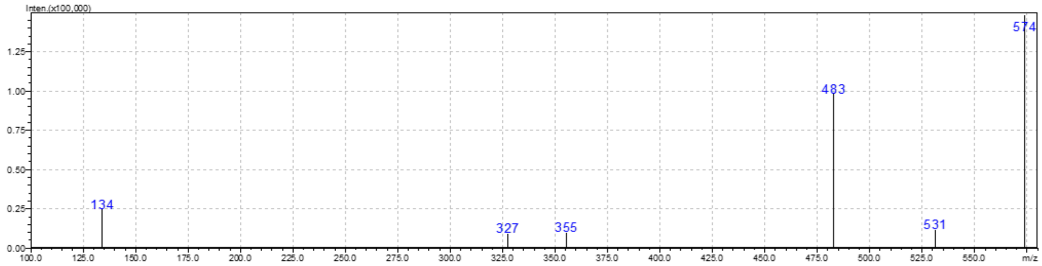

[MS Spectrum]

# of Peaks	6	
Raw Spectrum	[0.592] (scan:[238])	
Background	No Background Spectrum	
Precursor	574.00	
Base Peak	m/z 574.05 (Inten : 148,372)	
m/z	Absolute Intensity	Relative Intensity
134.15	24757	16.69
327.50	9662	6.51
355.35	10286	6.93
482.90	98195	66.18
531.25	12009	8.09
574.05	148372	100.00
Event	4	

Figure S37. The –ESI-MS spectrum of triazinone **4d**.
